# Temporal development and potential interactions between the gut microbiome and resistome in early childhood

**DOI:** 10.1128/spectrum.03177-23

**Published:** 2024-01-09

**Authors:** Lanlan Zhao, Xiao Yang, Yijia Liang, Ziyi Zhang, Yanwen Ding, Yihui Wang, Bin Chen, Jiacheng Wu, Chuandi Jin, Guoping Zhao, Ziyun Li, Lei Zhang

**Affiliations:** 1 MicrobiomeX, School of Public Health, Cheeloo College of Medicine, Shandong University, Jinan, China; 2 State Key Laboratory of Microbial Technology, Shandong University, Qingdao, China; 3 CAS Key Laboratory of Computational Biology, Bio-Med Big Data Center, Shanghai Institute of Nutrition and Health, University of Chinese Academy of Sciences, Chinese Academy of Sciences, Shanghai, China; University of Pretoria, Pretoria, South Africa

**Keywords:** early childhood, infant, metagenome, gut resistome, gut microbiome, antibiotics, antibiotic resistance genes

## Abstract

Antimicrobial resistance-associated infections have become a major threat to global health. The gut microbiome serves as a major reservoir of bacteria with antibiotic resistance genes; whereas, the temporal development of gut resistome during early childhood and the factors influencing it remain unclear. Moreover, the potential interactions between gut microbiome and resistome still need to be further explored. In this study, we found that antibiotic treatment led to destabilization of the gut microbiome and resistome structural communities, exhibiting a greater impact on the resistome than on the microbiome. The composition of the gut resistome at various developmental stages was influenced by the abundance and richness of different core microbes. First exposure to antibiotics led to a dramatic increase in the number of opportunistic pathogens carrying multidrug efflux pump encoding genes. Multiple factors could influence the gut microbiome and resistome formation. The data may provide new insights into early-life research.

**IMPORTANCE:**

In recent years, the irrational or inappropriate use of antibiotics, an important life-saving medical intervention, has led to the emergence and increase of drug-resistant and even multidrug-resistant bacteria. It remains unclear how antibiotic exposure affects various developmental stages of early childhood and how gut core microbes under antibiotic exposure affect the structural composition of the gut resistome. In this study, we focused on early antibiotic exposure and analyzed these questions in detail using samples from infants at various developmental stages. The significance of our research is to elucidate the impact of early antibiotic exposure on the dynamic patterns of the gut resistome in children and to provide new insights for early-life studies.

## INTRODUCTION

In recent years, there has been a gradual emergence and increase in antimicrobial-resistant bacteria, particularly multidrug-resistant (MDR) bacteria (1), which has led to antimicrobial resistance (AMR)-associated microbial infections becoming one of the leading causes of death worldwide. Approximately 4.95 million deaths were associated with bacterial AMR in 2019, including 1.27 million deaths attributable to bacterial AMR, of which approximately 20% were in children under 5 years of age (2). Almost one in seven deaths in children under 5 years of age is due to pneumonia, which is easily caused by recurrent bacterial infections of the respiratory tract (3). The duration of fever was longer in the pneumonia patients with AMR than those with no AMR, which led to longer hospitalizations in the former (4). Drug resistances of bacteria isolated from lower respiratory tract aspirations of children with ventilator-associated pneumonia were serious, and the antimicrobial resistance of G^-^ bacilli showed multiple drug resistance (5). Another study showed that *Streptococcus pneumoniae* isolates from children with severe community-acquired pneumonia were resistant to many antibiotics (6). AMR reduces the clinical efficacy of antibiotics, the mainstay of treatment for bacterial infections in children, and increases treatment costs.

The human gut microbiome plays a key role in human health and disease as a “super organ” throughout life, particularly in infancy (7). At the same time, it is a major reservoir of antibiotic resistance genes (ARGs) (8), and ARG-carrying commensal bacteria in the gut can transmit AMR to pathogens through horizontal gene transfer (HGT) under antibiotic selection. Enterobacteriaceae, especially *Escherichia coli*, as the major carriers or transmitters of AMR, colonize the gut of infants within a week of birth (9, 10). It has been investigated that the gut of infants contains high levels of ARGs, and high levels of ARGs have been detected even in the gut of infants not exposed to antibiotics (10–12).

Statistically, most children receive antibiotics before the age of 2 and a higher percentage receive antibiotics in the second year (13–15). It is estimated that in low- and middle-income countries, children receive an average of 11 antibiotic courses in the first 2 years of life (16). Multiple cross-sectional analysis studies have found that early antibiotic exposure not only leads to a decrease in gut microbial diversity in the infant gut microbiome (17, 18) but also significantly increases the diversity and abundance of ARGs in infants (19). Early antibiotic exposure and early antibiotic-resistant bacterial infections greatly affect the health and subsequent development of children (20).

According to Stewart et al. (21), the developing gut microbiome goes through three distinct phases of microbiome progression: a developmental phase (months 3–14), a transitional phase (months 15–30), and a stable phase (months 31–46). Moreover, evidence suggests that antibiotics may severely damage the host-microbiota ecosystem and delay normal gut flora colonization (22), which is related to subsequent diseases (23–26). Available data have suggested that antibiotic exposure disrupts the shaping of the gut microbiome before it reaches a stable stage (27, 28) and significantly affects the structural composition of the gut resistome (29, 30). However, it remains an open question as to how antibiotic exposure affects the intestinal resistome at different developmental phases in early childhood in a longitudinal birth cohort, and what are the core microbes that affect the resistome at different phases. In this study, we focused on early antibiotic exposure and analyzed these questions in detail using a sample of 212 infants at various developmental stages (0–3, 3–6, 6–12, 12–18, 18–36 months).

## RESULTS

### Early antibiotic exposure has a greater effect on the gut resistome than on the gut microbiome in children

**Fig 1:**
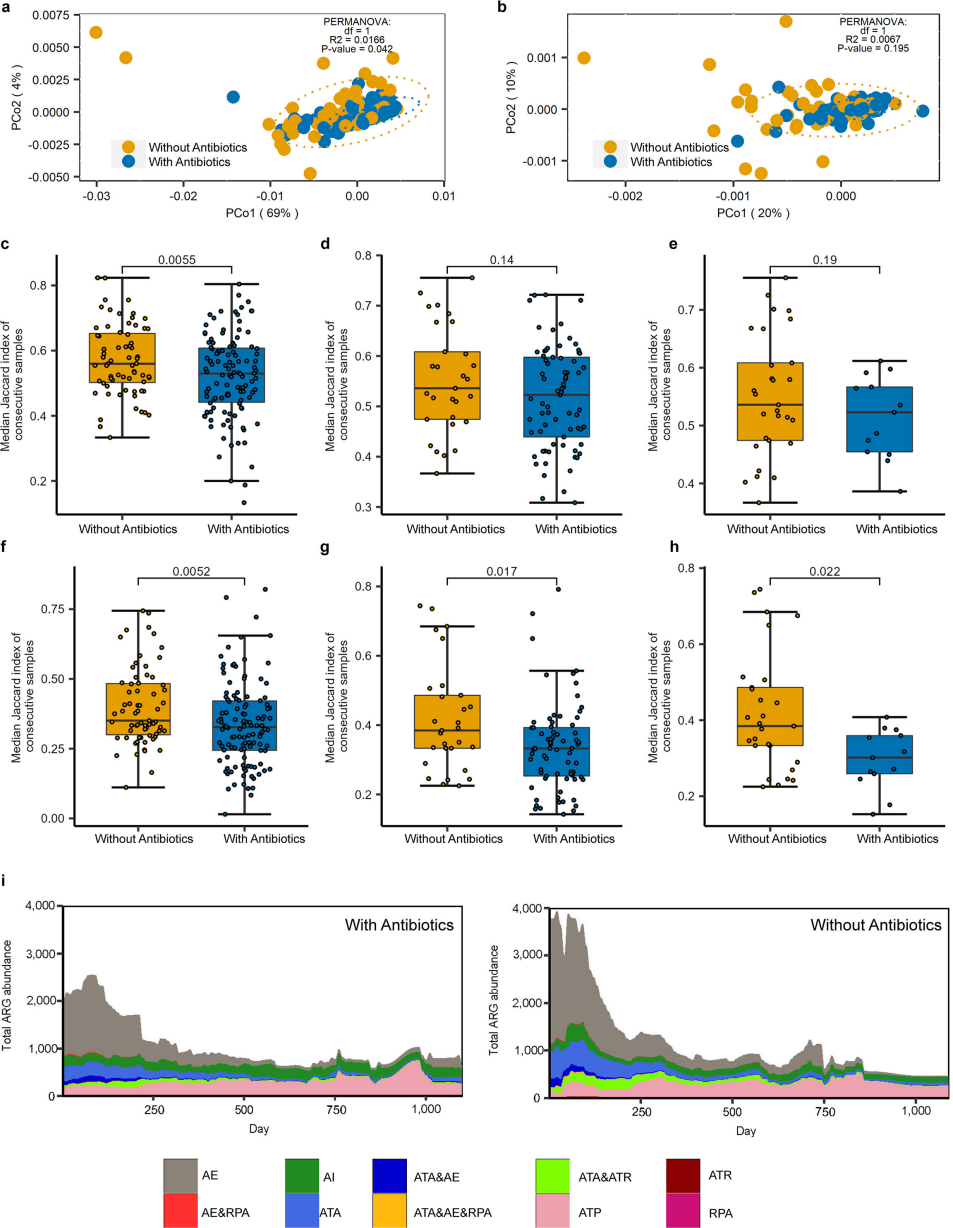
Effects of antibiotic treatment on gut microbiota and resistome in children. β-diversity analysis and PERMANOVA of (**a**) ARG profiles and (**b**) gut microbiota. Comparison of (**c**) gut microbiota and (**f**) gut resistome stability in children of “With Antibiotics” and “Without Antibiotics” groups, with at least two samples per child. Comparison of (**d**) gut microbiota and (**g**) gut resistome stability in children of “With Antibiotics” and “Without Antibiotics” groups, with at least three samples in different age groups per child. Comparison of (**e**) gut microbiota and (**h**) gut resistome stability in children of “With Antibiotics” and “Without Antibiotics” groups, with at least three samples in different age groups per child and number of antibiotic treatments no less than six in “With Antibiotics” children. *P* value is on the top of the picture in (**c–h**). *t* test was used for (**c–e**), and Wilcoxon rank-sum test was used for (**f–h**). (**i**) Average composition of total ARG abundance in gut of “With Antibiotics” and “Without Antibiotics” children during the first 3 years of age. AE, antibiotic efflux; AI, antibiotic inactivation; ATA, antibiotic target alteration; ATP, antibiotic target protection; ATR, antibiotic target replacement; and RPA, reduced permeability to antibiotic.

To analyze the effect of antibiotic treatment on gut microbiome and resistome, 185 of 212 children (with at least two metagenomic sequencing samples) were selected, 118 of whom received antibiotics (“With Antibiotics” group) and 67 children belonged to the “Without Antibiotics” group. The types of antibiotics used, duration, and specific clinical disease for antibiotics use of the 118 “With Antibiotics” children are listed in Table S1. As a result, there was a significant difference in the β-diversity of the gut resistome between the two groups (PERMANOVA, *P* = 0.042), but not in the gut microbiome (PERMANOVA, *P* = 0.195), suggesting that antibiotic treatment before the gut microbiome reaching a stable phase (21) has a higher effect on the gut resistome than on the gut microbiome (Fig. 1a and b). Notably, differences in children’s country of origin did not affect the results of the β-diversity analysis (Fig. S1). Next, we constructed an *in vitro* community using four strains of *E. coli*, of which *E. coli* DH5α (pRP4) was an ampicillin-resistant strain and the remaining three strains were ampicillin- sensitive. The use of M9 medium ensured that the total number of *E. coli* remained constant before and after 1 h (Fig. S2a), and the proportion of ampicillin-resistant *E. coli* was increased at different concentrations of ampicillin selection pressure compared with the control (Fig. S2b). The above results suggested that in early life, antibiotics may increase the survival advantage of antibiotic-resistant bacteria without altering the composition of the gut microbiome and help them occupy ecological niches of antibiotic-sensitive bacteria in the same species, which in turn may significantly alter the composition of the gut resistome.

### Early antibiotic exposure leads to a more structurally unstable gut microbiome and gut resistome

To explore the effect of antibiotics on the stability of gut microbiome and resistome, we used the median of the Jaccard index at continuous time points as the overall stability index for each child. We excluded subjects with only one sample and divided the infants into “With Antibiotics” and “Without Antibiotics” groups. We found that the “With Antibiotics” group had significantly lower community stability, both for gut microbiome and resistome (Fig. 1c and f) (*t* test, *P* = 0.0055, and Wilcoxon rank-sum test, *P* = 0.0052, respectively). Subsequently, we screened the subjects with at least three samples at different age groups and found that antibiotic-treated infants still had significantly lower structural stability of resistome and that their gut microbial community stability was lower but not significant (Fig. 1d and g) (Wilcoxon rank-sum test, *P* = 0.017, and *t* test, *P* = 0.14, respectively). Furthermore, we screened the “With Antibiotics” children with antibiotic treatments of no less than six times and found that these children still had significantly lower structural stability of resistome (Fig. 1e and h) (Wilcoxon rank-sum test, *P* = 0.022). Moreover, all children in the antibiotic-treated group had a higher variability of the stability index per child compared with the non-antibiotic-treated group (Fig. S3). Considering the age of the sample, we screened the subjects with samples at different age groups (0–6, 6–12, and 18–36 months) and found that the antibiotic-treated infants still had significantly lower structural stability of resistome (Fig. S4a, *t* test, *P* = 0.049) and that their gut microbial community stability was lower but not significant (Fig. S4b, *t* test, *P* = 0.81).

### First antibiotic exposure leads to a sharp increase in the abundance of opportunistic pathogen carrying multidrug efflux pump encoding gene

To investigate the longitudinal variation of antibiotic treatment on the abundance of ARGs, we divided all infants into “With Antibiotics” and “Without Antibiotics” groups and plotted longitudinal trajectories of ARG abundance. We found that the total abundance of ARGs was higher in the “With Antibiotics” group at about 3 years of age (Fig. 1i) (Wilcoxon rank-sum test, *P* = 0.1062). Further analysis revealed that antibiotic target protection (ATP) was the main mechanism of bacterial antibiotic resistance in the “Without Antibiotics” group (Fig. 1i). However, the mechanisms were more abundant in the “With Antibiotic” group (Fig. 1i), including antibiotic efflux (AE), antibiotic inactivation (AI), and ATP, where AI was mainly a function of the gene encoding β-lactamase (Fig. S5). Notably, we achieved a rapid weakening of ARG abundance throughout approximately the first 250 days of life, regardless of whether the infant received antibiotics, mainly by reducing the abundance of the antibiotic efflux pump genes (Fig. 1i). ARG abundance with the mechanism of AE was highest in the first 3 months of life, with a decreasing trend consistent with Proteobacteria, and further analysis revealed that these ARGs were predominantly of *E. coli*, regardless of age (Fig. S6 and S7).

**Fig 2:**
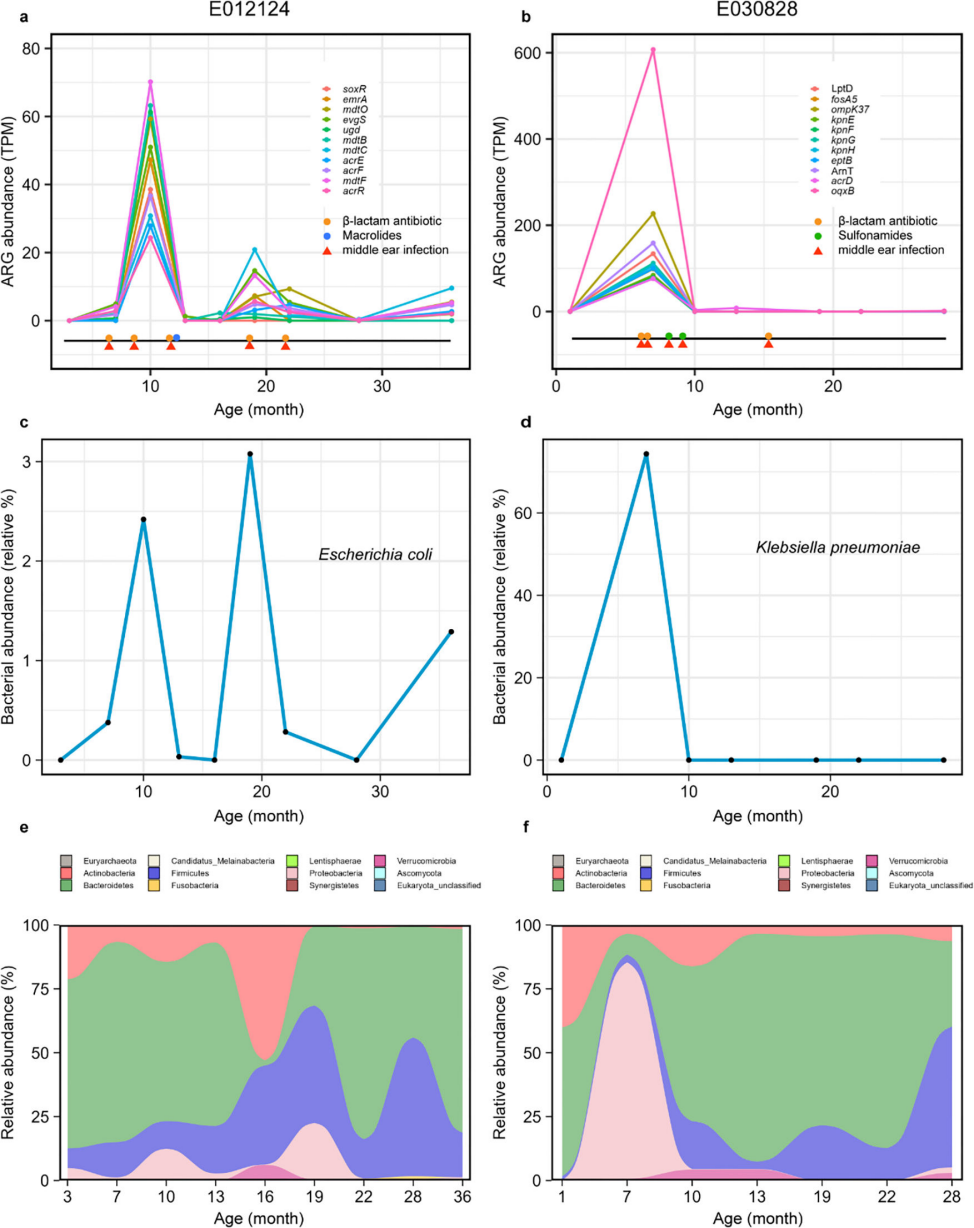
ARG, species, and average phylum-level composition profiles. (**a, b**) Abundance of ARG (TPM, transcripts per kilobase million) in two children over time, together with the timing of individual antibiotic courses (colored dots) and diseases (colored triangles). (**c, d**) Relative abundance of species that most correlated with the ARG profiles (**a, b**). (**e, f**) Average phylum-level composition of two children.

Next, we analyzed the changes in the abundance of ARG profiles, bacteria species, and composition of gut microbiome at the individual level during disease onset and antibiotic exposure. To ensure continuity of visualization, three children (E030828, E012124, and E022960) who had received no less than five times of antibiotics and no less than seven samples from at least four age groups were finally identified as subjects for the study (Fig. S8). We could clearly observe that the ARG abundance increased rapidly during antibiotic treatment and decreased extremely rapidly after antibiotic withdrawal, and in each case, changes in the abundance of an opportunistic pathogen were strongly correlated with this trend, indicating that they are the likely harbor of ARGs (Fig. 2; Fig. S9). In a notable example (subject E030828), we detected an increase in the relative abundance of *Klebsiella pneumoniae* and the proportion of Proteobacteria at 7 months of amoxicillin treatment (Fig. 2d and f). The same trend of increase occurred in the abundance of *oqxB* (AE-encoding gene) and *ompK37* gene (encoding a porin-protein resistant to β-lactams) (Fig. 2b). Similar results were observed in subject E012124 (Fig. 2a); the increasing trend of ARGs was tightly correlated with *E. coli*, which is annotated as the host of these genes (Fig. 2c). The first exposure to antibiotics led to an increase in the abundance of opportunistic pathogen carrying multidrug efflux pump encoding gene in early life (Fig. 1i, Fig. 2a and b).

### Multiple factors influence the shape of gut microbiome and resistome in early childhood and further influence the host antibody production level

**Fig 3:**
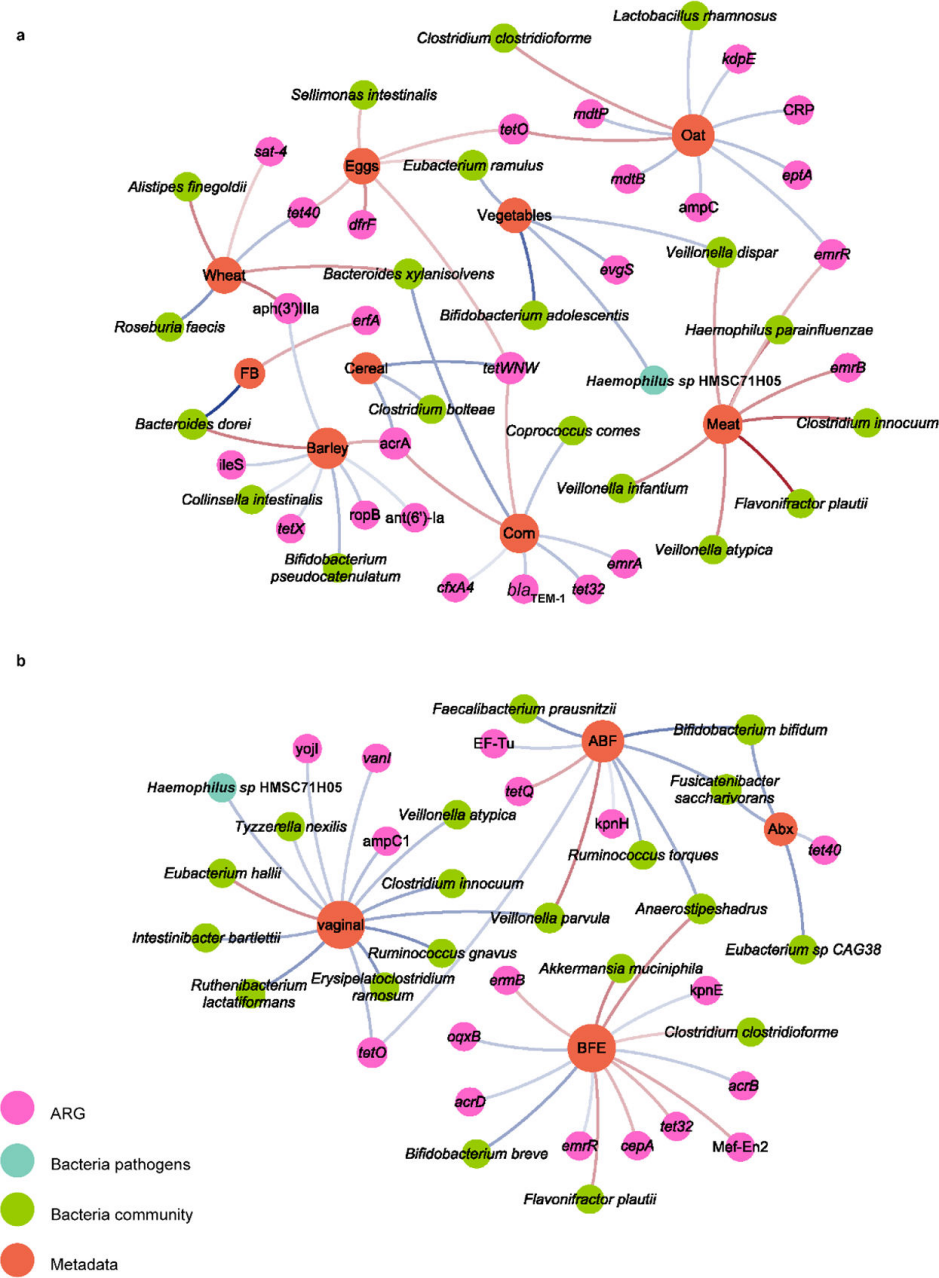
Network of metadata and ARGs or bacteria. Correlations between ARG or bacteria and (**a**) dietary compounds and (**b**) other metadata. Correlations between metadata and ARG or bacteria were calculated through MaAsLin2 analysis. Only statistically significant correlations (*P* < 0.05) were plotted. Pink, blue, green, and orange circles represent ARGs, bacterial pathogens, bacterial community, and metadata, respectively. The color intensity of connective lines is proportional to the correlation coefficient, in which blue line and red line indicate negative and positive correlation, respectively. ABF, any baby formula; Abx, after antibiotic; BFE, breastfeeding end; and FB, fruits and berries.

Next, we investigated and found significant correlations (*P*_FDR_ <0.25) between multiple factors and bacterial species or ARGs, especially in early childhood with different dietary profiles (Fig. 3). We observed that vegetables and coarse grains (corn, cereal, oat, and barley) were significantly negatively correlated with ARGs, such as β-lactam drug-resistant genes *ampC* and *cfxA4*. In particular, the level of vegetable consumption was significantly negatively correlated with the pathogen *Haemophilus* sp. HMSC71H05 (Fig. 3a; Table S2). In contrast, meat, fish, eggs, and refined grains (wheat and rice) were positively associated with some pathogens or opportunistic pathogens such as *Haemophilus parainfluenzae* and *Clostridium perfringens* (Fig. 3a; Table S2). Among these factors, we found an increase in *Eubacterium hallii* during vaginal delivery, which contributed to the formation of intestinal short-chain fatty acids (SCFAs) (31), and a decrease in opportunistic pathogens and ARGs such as AmpC1-β-lactamase encoding gene, tetracycline-resistant gene *tetO*, and vancomycin-resistant gene *vanI* (Fig. 3b; Table S2). Multiple factors, especially dietary structure, significantly influence the gut microbial composition and abundance of ARGs in early childhood, which may have an impact in the shaping of the gut microbiome and resistome.

Next, we explored whether the gut microbiota mediated the influence of the use of antibiotics on host antibody production level (IgE level). We observed that the log10 IgE level of “With Antibiotics” children was higher although not significant (Without Antibiotics: 0.78 ± 0.8, With Antibiotics: 0.958 ± 0.64, *t* test, *P* = 0.16). Then, we found 416 bacteria with significant differences between the “With Antibiotics” and “Without Antibiotics” groups, including 254 at species level (Wilcoxon rank-sum test, *P*_FDR_ <0.05), and antibiotic treatment increased the IgE level mainly through bacteria as mediators (Table S3). Notably, most of the mediating bacteria belong to Firmicutes, with the largest proportion of *Clostridiales* and *Lactobacillales*, and the mediating effects performed by species belonging to the same genus may be variable (Table S3). Further analysis revealed that the existence of core microbes such as *Clostridiales*, *Lactobacillales*, and *Actinomycetales* may play a key role in the process by which factors such as antibiotics influence IgE level.

### Structural compositions of gut resistome at different phases of early childhood were influenced by the richness and abundance of specific core microbes

**Fig 4:**
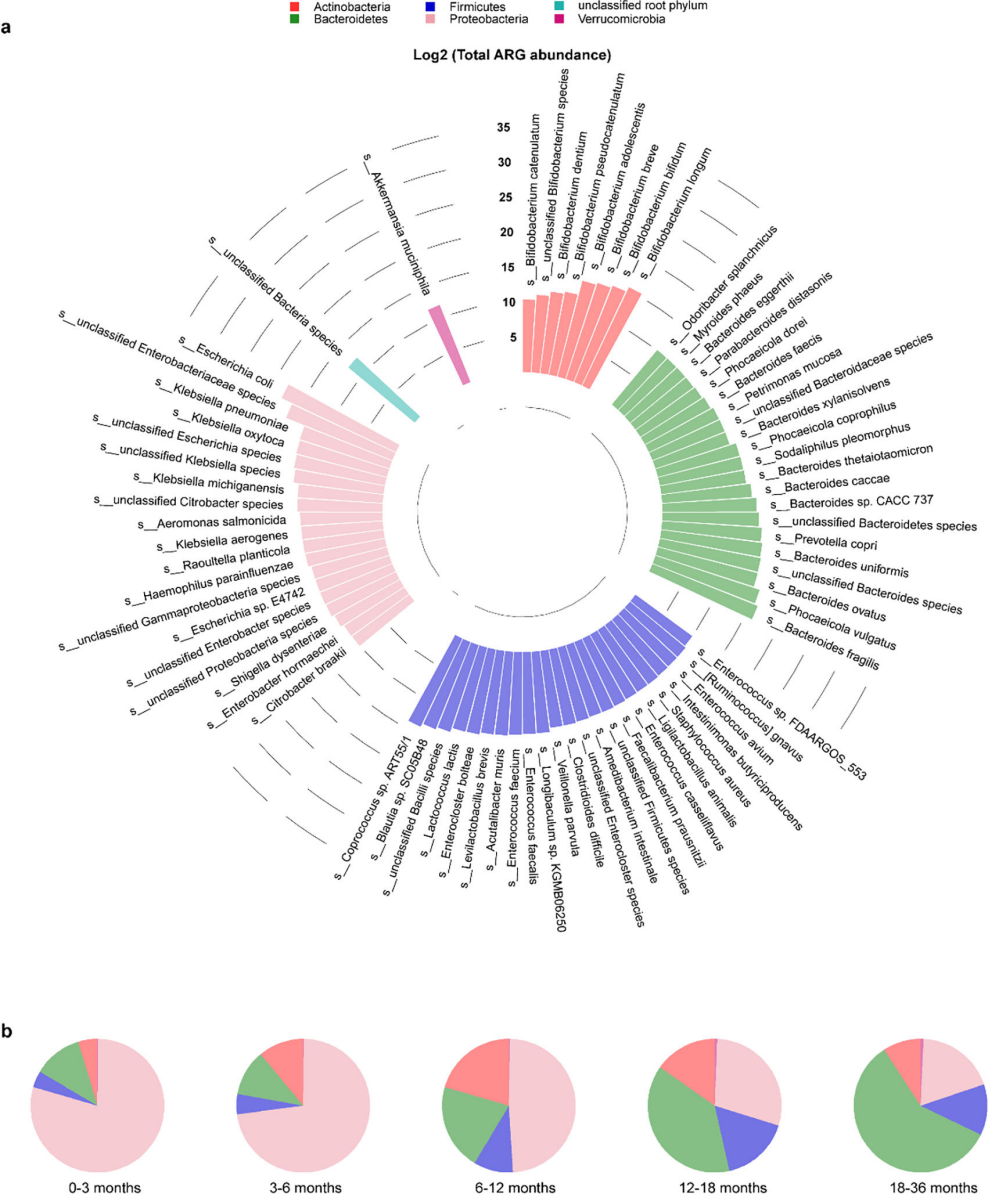
Distribution of ARGs among bacterial species and phyla from the gut of children. (**a**) Log-transformed total ARG abundance among bacterial species and phyla. To facilitate viewing, only those species with log-transformed total ARG abundance ARGs of no less than 10 are shown. (**b**) Average phylum-level ARG abundance in five age groups.

**Fig 5:**
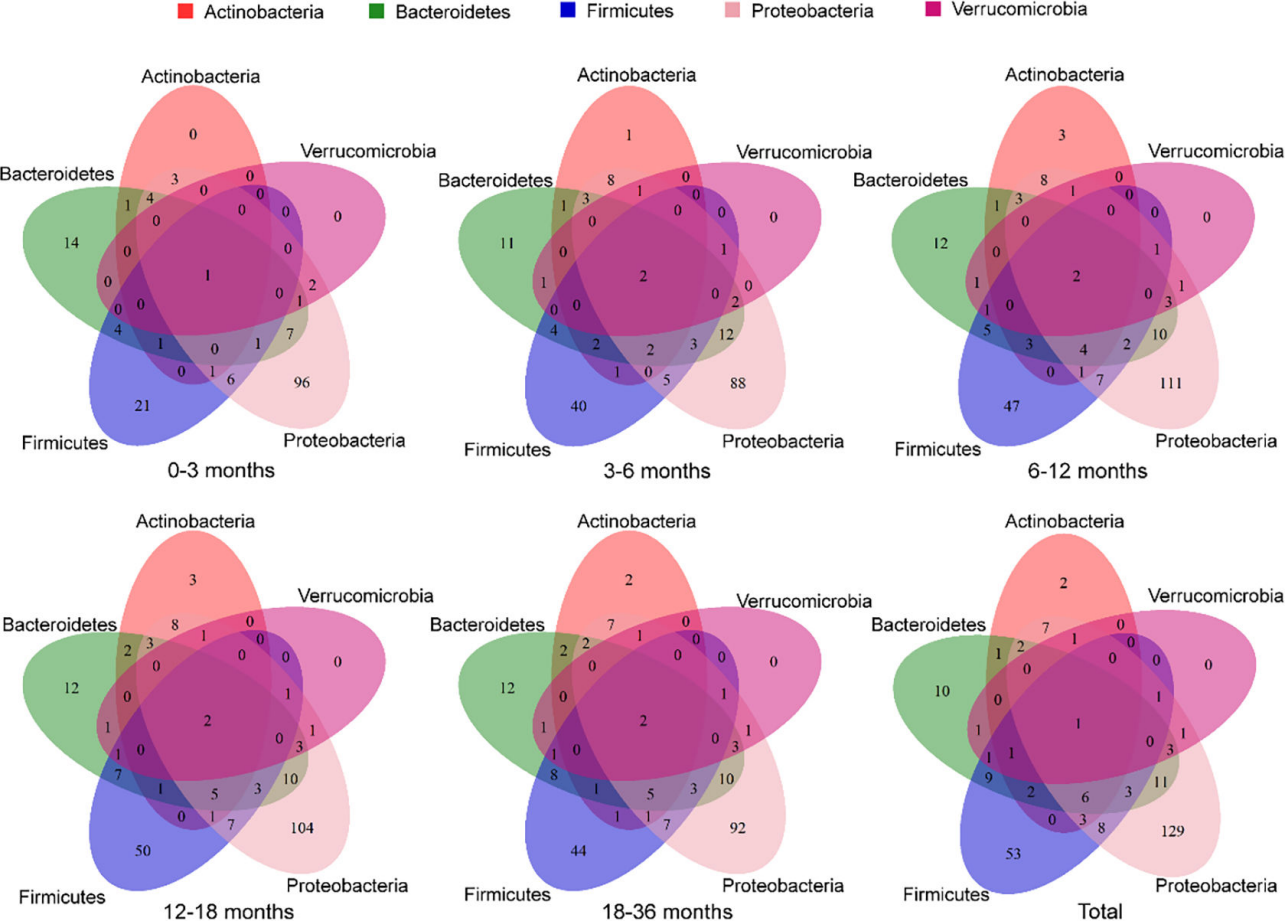
Venn diagram of the numbers of overlapping and unique ARGs. Venn diagrams are presented from five age groups and total.

To characterize ARGs from different bacteria in the infant gut, we traced the origin of each gene using annotated results of the metagenomic contigs. In the samples of overall and different ages (0–3, 3–6, 6–12, 12–18, 18–36 months), ARGs were traced back to 166, 92, 120, 153, 150, and 148 species, respectively (Fig. S10 to S14). It was noteworthy that the species of Proteobacteria, such as *E. coli* and *Klebsiella*, contributed the most ARG richness at overall level (Fig. 4a; Fig. S15); in detail, Proteobacteria had 129 unique ARGs overall and ranked first in all age groups (Fig. 5). There were significant differences in the proportion of average ARG abundance at phylum-level in five age groups, especially among the first group and the last three groups (Fisher’s exact test, *P*_FDR_ <0.05) (Fig. 4b). Notably, Proteobacteria had the highest average abundance of ARGs in the first three age groups, but the average ARG abundance of Bacteroidetes was higher than that of Proteobacteria in the latter two age groups (Fig. S16 to S22).

**Fig 6:**
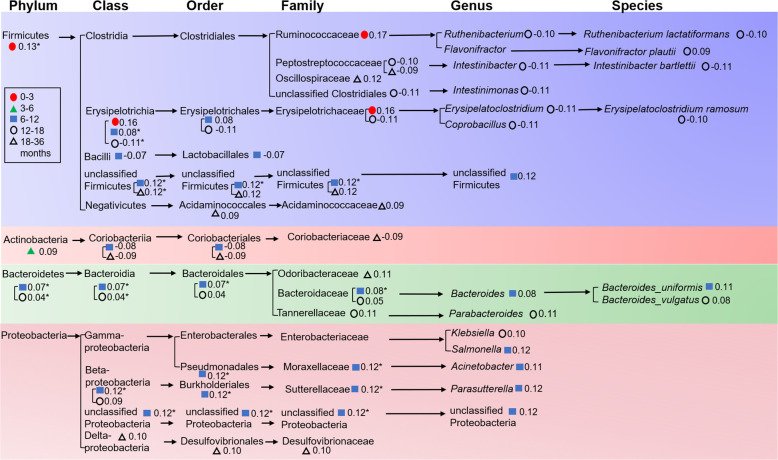
The gut core microbes related to gut resistome in each age group from phylum to species. Only statistically significant correlations (*P*_FDR_ <0.25) were plotted. *: *P*_FDR_ <0.05.

To explore the correlation between the gut microbiome and the gut resistome for each age group, we performed the analysis using Procrustes analysis, Mantel test, and co-occurrence network analysis. The Procrustes analysis confirmed that the correlation between the composition of the microbial community and the ARG profile varied with age, showing a significant correlation only at 3–6 months (M^2^ = 0.92, *P* = 0.01, Table S4). *E. coli* and *K. pneumoniae* were significantly correlated (*P* < 0.05) with the ARG profiles (Fig. S23). As shown in Fig. 6, we found that most of the gut core microbes related to gut resistome (*P*_FDR_ <0.25) in each age group belonged to Firmicutes, especially because only Firmicutes bacteria were present at 0–3 months. In particular, 6–12 months had the highest number of core microbes that significantly affected the structure of the gut resistome (Fig. 6). All these results suggested that the gut resistome was most significantly influenced by core microbes in early childhood, especially around the first 6 months of early life (Fig. 4 to 6; Table S4)—a period when the use of antibiotics, especially β-lactams, promoted the increased abundance of opportunistic pathogens such as *Klebsiella* and *Escherichia* (Fig. 2). These Enterobacteriaceae not only often carry ARGs on their chromosomes that encode multidrug efflux pumps (e.g., *tolC*, *ompK37*, etc.) but can also act as transmitters or reservoirs of ARGs that can move with the help of mobile genetic elements such as plasmid (10, 32, 33).

## DISCUSSION

With the development of metagenomic sequencing technology, there is an increasing interest in the human gut microbiome (34, 35), especially that of infants, which may contribute to the fundamental development of the immune system. Analyzing the correlation between gut microbes and disease development only at the level of microbial structure changes may neglect many important information, such as the ARG profiles and phage profiles carried by these bacteria, which may change with the adjustment of microbial structure, and the impact of such changes on the host organism is often overlooked. In this study, we conducted further analysis of data obtained from metagenomic sequencing in a longitudinal birth cohort (36) to explore in depth how antibiotic exposure affects the gut resistome at different developmental phases in early childhood and what are the specific core microbes that influence the gut resistome at different phases.

From the analysis of longitudinal data of gut microbiome and resistome in early life, the effect of antibiotics was more significant in the gut resistome (Fig. 1) because selection pressure caused by antibiotic exposure accelerated the overall selection of antimicrobial-resistant bacteria carrying ARGs. The abundance of bacteria containing specific ARGs increased after the corresponding antibiotic treatment (Fig. 2; Fig. S2), which indicated that antibiotics may increase the survival advantage of antibiotic-resistant bacteria without altering the composition of the gut microbiome, which in turn may significantly alter the gut resistome. Furthermore, the results of this study are consistent with other related studies (23) in which early exposure to antibiotics led to disruptions in the structure and stability of both the gut microbiome and the gut resistome.

In the longitudinal analysis, we also found that the abundance of ARGs was highest in the first year of life, especially in the first 6 months, which may be related to changes in the dominant bacteria in the infant gut, which may be diet related. The analysis found that different diets were significantly associated with the abundance of specific bacteria in the gut and that factors such as breastfeeding and mode of delivery were able to significantly influence the composition of infant’s gut microbiota and ARG profiles. An animal study also found that a reduction in breastfeeding was associated with a decrease in ARG abundance (37). Recent studies have found that early lifestyle influences the development of gut resistome (34, 38, 39), and our results further highlight the importance of dietary structure in early life in building the gut ecology of children.

Our results suggested that multidrug efflux is a major mechanism of AMR in early life, especially before 6 months, regardless of antibiotic use (Fig. 1i, Fig. 2; Fig. S9), which may be a strategy of bacterial self-defense that would help delay bacterial survival and contribute to bacterial AMR production (40). Further analysis revealed that there were certain rules in bacteria-carrying ARGs, which may be related to the dominant bacteria in different periods. This is consistent with a previous cross-sectional study (33) in which Proteobacteria contributed the most abundant and unique ARGs in 1-year-old children. Although the abundance and richness of ARGs carried by Firmicutes was not the highest in each age group, the growth pattern of Firmicutes bacteria early in life and the impact of core microbes in the Firmicutes on the gut resistome are noteworthy. A recent study found that the increase in the abundance of several genera in Firmicutes around the age of 1 year was related to the lack of breastfeeding at the time of sampling (38). The importance of Firmicutes was also reflected in studying the mediating role of bacteria in the increase of IgE levels caused by antibiotic treatment; we found that most bacteria belong to Firmicutes. Moreover, it is worth noting that the effects of different species may be quite different even if they belong to the same family or even genus (41, 42).

Since gene expression is usually more representative of functional activity than gene content, a comprehensive metatranscriptome analysis can provide additional knowledge about the function and regulation of resistance genes (43, 44). It should be noted that due to the limitations of using open source data, we identified the presence of ARGs but could not determine whether these genes were functional. Therefore, further improvements, such as functional metagenomics or functional assays, would help assess the actual antibiotic resistance capabilities of the gut microbiota in future studies.

Overall, our study explored the dynamic patterns of the gut microbiome and resistome at different development phases in early childhood, analyzed the potential correlations between the two, and analyzed the specific core microbes that influence the gut resistome at different developmental stages. This study elucidated the impact of early antibiotic exposure on the dynamic patterns of the gut resistome in children and provided new insights for early-life studies. It also demonstrated that the longitudinal cohort data analysis of the gut in early childhood requires the combination of microbiome and resistome for in-depth exploration.

## MATERIALS AND METHODS

### Analysis of metagenomic samples

In this study, we retrieved the metagenomic sequencing data of the DIABIMMUNE cohort from the National Center for Biotechnology Information (NCBI) database (BioProject: PRJNA290380) and collected the corresponding metadata based on the article by Bonder et al. (36). In the DIABIMMUNE cohort, infants from each country (Russia, Estonia, and Finland) were selected on the basis of similar histocompatibility leukocyte antigen (HLA) risk class distribution and matching gender. The information of samples and antibiotic exposure in the cohort are shown in Fig. S8. Ultimately, 783 metagenomic sequencing data were certified by MD5 and assessed for sequencing reads quality by FastQC and MultiQC (45) software. Reads quality were controlled using the KneadData pipeline (using trimmomatic options “ILLUMINACLIP: NexteraPE-PE.fa.fa:2:40:15 SLIDINGWINDOW:4:20 MINLEN:50” and other default settings), and quality-controlled samples were taxonomically and functionally profiled using MetaPhlAn3 (46) and HUMAnN3 (47) with default settings, respectively. The median reads count of quality-controlled samples was 2567262 (interquartile range: 1614455, 3810102). Then, high-quality reads were assembled into contigs using MEGAHIT (48) with default settings. To determine the metagenomic signature of ARGs, open reading frames (ORFs) were predicted using Prodigal (49), and the redundancy in the ORF-predicted results was eliminated by CD-HIT (50) (using the setting “-aS 0.9 -c 0.95 -G 0 -g 0 -T 20 -M 0”) to construct the non-redundant gene catalog, which was quantified in TPM (transcripts per kilobase million) using the Salmon tool with default settings. To estimate the taxonomic profiles and functional annotations of the non-redundant gene catalog, the contigs files were annotated against the NCBI nt database using Kraken2 (51) and protein sequences were aligned against the CARD (52) database using DIAMOND (53) with ‘--clean’ option.

### Analysis of the average microbial composition and ARG abundance in the gut of children

Country-level (Russia, Estonia, and Finland) and antibiotic-level (antibiotic treatment or not) average microbial profiles and ARG abundance were generated using the sliding window averaging analysis. Average phylum composition and ARG abundance were computed in a sliding window of size 90 days, with a step size of 5 days. The alluvial figures were drawn by ggalluvial package (0.12.3).

### Beta-diversity analysis

The Bray-Curtis distance metric of microbial taxonomy and ARGs was calculated by PLdist using pldist package in R (v1.0.0.0000), dimensionality was reduced by principal coordinates analysis (PCoA) using APE package in R (v5.6-1), grouping factors were tested by PERMANOVA with a default setting using the Vegan package in R (v2.5-7), and graphs were visualized using the ggplot2 package (v3.3.5). PLdist analysis is an analysis for ecological dissimilarities for paired and longitudinal moicrobiome.

### Analysis of stability

Jaccard similarity index was calculated for all consecutive samples of each child using stats package (v4.2.0), then the medium Jaccard index for each child was reported and plotted for “With Antibiotics” and “Without Antibiotics” children. Considering the sample age, we screened the subjects with samples at different age groups (0–6, 6–12, and 18–36 months). Medium Jaccard index for each child in “With Antibiotics” and “Without Antibiotics” children was tested using Wilcoxon rank-sum test or *t* test. Boxplot graphics were created using the R package ggplot2 (v3.3.5) and RColorBrewer (v1.1-3). Box boundaries were the 25th and 75th percentiles, and the median was highlighted.

#### *In vitro* experiments on structural changes of *E. coli* flora under antibiotic stress

In this study, four strains of *E. coli* [DH5α (pRP4), MG1655, BL21 (pET28a-*gfp*), and BL21 (pET28a-*rfp*)] were used to construct *in vitro E. coli* flora (the number of each strain was about 10^8^ CFU and the mixing ratio was 1:1:1:1). *E. coli* DH5α (pRP4) was an ampicillin-resistant strain and the remaining three strains were ampicillin-sensitive. These four strains were all from our laboratory collection (Fig. S2a), and the proportion of ampicillin-resistant *E. coli* was increased at different concentrations of ampicillin selection pressure compared with the control (Fig. S2b). The β-lactam antibiotic ampicillin was used as an antibiotic stress (final concentrations of 0, 2, 4, and 10 mg/L, respectively) with a treatment time of 1 h. The use of M9Ca medium ensured that the total number of *E. coli* remained constant before and after 1 h. The detailed steps were described in our previous study (54).

### Mediation analysis

We selected each child’s last measured IgE level and metagenomic data sample prior to the time of IgE measurement, and adjusted them for country and respective collection times using snm package (v1.42.0). We screened subjects with no antibiotics after the metagenomic sequencing sample collection time. Wilcoxon rank-sum test and false discovery rate (FDR) correction were performed on the corrected microbial data. The mediator variable was the significantly differential microbes, the independent variable was whether a child received antibiotics, and the dependent variable was log10 IgE level. We used the R package Mediation (v4.5.0) for mediation analysis and we tested for statistical significance using bootstrap approach (100 iterations, α = 0.05).

### Analysis of differential species and ARGs

Associations between clinical metadata and microbial taxon abundances or ARG abundances in all samples were tested using Maaslin2 package (v1.8.0). The following variables were used as random effects in all models: subject ID and sequencing batch ID (whole-genome shotgun). Furthermore, allergy and IgE measurements were available for only a subset of subjects and were tested using a separate model with fewer samples.

The network was constructed by using Cytoscape (3.9.0). In the network, each edge denotes a significant correlation (*P*_FDR_ <0.25). A different color of a node indicates the metadata, ARGs, bacterial pathogens, and bacterial community, and the size of a node is proportional to the number of significant interactions between them. The color of one edge indicates the correlation between them in positive or negative.

### Analysis of ARGs in bacterial phylum

The observed ARG richness indicates the ARG category among different bacterial phyla in different age groups. Log2 (total ARG abundance) means the ARG abundance among different bacterial phyla in different age groups was added and log-transformed. A circular barplot was drawn using ggplot2 package (3.3.5). The Venn diagram of the numbers of overlapping and unique ARGs among five major bacterial phyla was visualized using VennDiagram package (1.7.1) and Venn package (1.10). Each child’s temporal trend of the ARG abundance and bacterial relative abundance was plotted on line charts using the R package ggplot2 (v3.3.5.). Average phylum-level ARG abundance or richness among five age groups was compared using chi-squared test or Fisher’s exact test, multiple comparisons, and FDR correction by rcompanion package (2.4.15) in R.

### Analysis of core bacteria related to gut resistome

At each microbial classification level, Mantel test was conducted for single bacteria and resistome data at the same age group to obtain core bacteria related to the whole antibiotic resistance gene data with FDR correction. Procrustes analysis was used for calculating the correlation between the composition of the microbial community and the ARG profile.

## Data Availability

The genome sequence data in this study are available in the NCBI BioProject database (https://www.ncbi.nlm.nih.gov/bioproject/) under accession number PRJNA290380.

## SUPPLEMENTAL MATERIAL

Figure S8 spectrum.03177-23-s0001.pdf The information of samples and antibiotic exposure in the cohort.

Supplemental material spectrum.03177-23-s0002.pdf Figures S1 to S23; Table S4.

Table S1 spectrum.03177-23-s0003.xlsx Types of antibiotics used, duration, and specific clinical disease for antibiotic use of 118 “With Antibiotics” children.

Table S2 spectrum.03177-23-s0004.xlsx Maaslin result of total samples.

Table S3 spectrum.03177-23-s0005.xlsx Mediation result with an acem value greater than zero and a *P* value less than 0.05.

ASM does not own the copyrights to Supplemental Material that may be linked to, or accessed through, an article. The authors have granted ASM a non-exclusive, world-wide license to publish the Supplemental Material files. Please contact the corresponding author directly for reuse.

## References

[1] McEwen SA, Collignon PJ. 2018. Antimicrobial resistance: a one health perspective. Microbiol Spectr 6. doi:10.1128/microbiolspec.ARBA-0009-2017 PMC11633550 29600770

[2] Antimicrobial Resistance Collaborators. 2022. Global burden of bacterial antimicrobial resistance in 2019: a systematic analysis. Lancet 399:629–655. doi:10.1016/S0140-6736(21)02724-0 35065702 PMC8841637

[3] Klein EY, Van Boeckel TP, Martinez EM, Pant S, Gandra S, Levin SA, Goossens H, Laxminarayan R. 2018. Global increase and geographic convergence in antibiotic consumption between 2000 and 2015. Proc Natl Acad Sci U S A 115:E3463–E3470. doi:10.1073/pnas.1717295115 29581252 PMC5899442

[4] Yuan C, Min F-M, Ling Y-J, Li G, Ye H-Z, Pan J-H, Wang L, Xie Y-P. 2019. Clinical characteristics and antibiotic resistance of mycoplasma pneumoniae pneumonia in hospitalized Chinese children. CCHTS 21:749–754. doi:10.2174/1386207322666190111112946 30636596

[5] Cai X-F, Sun J-M, Bao L-S, Li W-B. 2011. Distribution and antibiotic resistance of pathogens isolated from ventilator-associated pneumonia patients in pediatric intensive care unit. World J Emerg Med 2:117–121. doi:10.5847/wjem.j.1920-8642.2011.02.007 25214995 PMC4129699

[6] Tran-Quang K, Nguyen-Thi-Dieu T, Tran-Do H, Pham-Hung V, Nguyen-Vu T, Tran-Xuan B, Larsson M, Duong-Quy S. 2023. Antibiotic resistance of Streptococcus pneumoniae in vietnamese children with severe pneumonia: a cross-sectional study. Front Public Health 11:1110903. doi:10.3389/fpubh.2023.1110903 37383272 PMC10294427

[7] Sommer F, Bäckhed F. 2013. The gut microbiota--masters of host development and physiology. Nat Rev Microbiol 11:227–238. doi:10.1038/nrmicro2974 23435359

[8] Le Blay G, Rytka J, Zihler A, Lacroix C. 2009. New in vitro colonic fermentation model for Salmonella infection in the child gut. FEMS Microbiol Ecol 67:198–207. doi:10.1111/j.1574-6941.2008.00625.x 19087202

[9] Lynch MDJ, Neufeld JD. 2015. Ecology and exploration of the rare biosphere. Nat Rev Microbiol 13:217–229. doi:10.1038/nrmicro3400 25730701

[10] Shao Y, Forster SC, Tsaliki E, Vervier K, Strang A, Simpson N, Kumar N, Stares MD, Rodger A, Brocklehurst P, Field N, Lawley TD. 2019. Stunted Microbiota and opportunistic pathogen colonization in caesarean-section birth. Nature 574:117–121. doi:10.1038/s41586-019-1560-1 31534227 PMC6894937

[11] Pärnänen K, Karkman A, Hultman J, Lyra C, Bengtsson-Palme J, Larsson DGJ, Rautava S, Isolauri E, Salminen S, Kumar H, Satokari R, Virta M. 2018. Maternal gut and breast milk Microbiota affect infant gut antibiotic resistome and mobile genetic elements. Nat Commun 9:3891. doi:10.1038/s41467-018-06393-w 30250208 PMC6155145

[12] Li X, Stokholm J, Brejnrod A, Vestergaard GA, Russel J, Trivedi U, Thorsen J, Gupta S, Hjelmsø MH, Shah SA, Rasmussen MA, Bisgaard H, Sørensen SJ. 2021. The infant gut resistome associates with E. coli, environmental exposures, gut microbiome maturity, and asthma-associated bacterial composition. Cell Host Microbe 29:975–987. doi:10.1016/j.chom.2021.03.017 33887206

[13] Aris IM, Lin P-I, Rifas-Shiman SL, Bailey LC, Boone-Heinonen J, Eneli IU, Solomonides AE, Janicke DM, Toh S, Forrest CB, Block JP. 2021. Association of early antibiotic exposure with childhood body mass index trajectory milestones. JAMA Netw Open 4:e2116581. doi:10.1001/jamanetworkopen.2021.16581 34251440 PMC8276083

[14] Resi D, Milandri M, Moro ML, Emilia Romagna Study Group On The Use Of Antibiotics In Children. 2003. Antibiotic prescriptions in children. J Antimicrob Chemother 52:282–286. doi:10.1093/jac/dkg302 12865400

[15] Hellman J, Grape M, Ternhag A. 2015. Antibiotic consumption among a Swedish cohort of children born in 2006. Acta Paediatr 104:1035–1038. doi:10.1111/apa.13097 26109274

[16] Fink G, D’Acremont V, Leslie HH, Cohen J. 2020. Antibiotic exposure among children younger than 5 years in low-income and middle-income countries: a cross-sectional study of nationally representative facility-based and household-based surveys. Lancet Infect Dis 20:179–187. doi:10.1016/S1473-3099(19)30572-9 31843383

[17] Reyman M, van Houten MA, Watson RL, Chu MLJN, Arp K, de Waal WJ, Schiering I, Plötz FB, Willems RJL, van Schaik W, Sanders EAM, Bogaert D. 2022. Effects of early-life antibiotics on the developing infant gut microbiome and resistome: a randomized trial. Nat Commun 13:893. doi:10.1038/s41467-022-28525-z 35173154 PMC8850541

[18] Rooney AM, Timberlake K, Brown KA, Bansal S, Tomlinson C, Lee K-S, Science M, Coburn B. 2020. Each additional day of antibiotics is associated with lower gut anaerobes in neonatal intensive care unit patients. Clin Infect Dis 70:2553–2560. doi:10.1093/cid/ciz698 31367771 PMC7286368

[19] Lebeaux RM, Coker MO, Dade EF, Palys TJ, Morrison HG, Ross BD, Baker ER, Karagas MR, Madan JC, Hoen AG. 2021. The infant gut resistome is associated with E. coli and early-life exposures. BMC Microbiol 21:201. doi:10.1186/s12866-021-02129-x 34215179 PMC8252198

[20] Bryce A, Hay AD, Lane IF, Thornton HV, Wootton M, Costelloe C. 2016. Global prevalence of antibiotic resistance in paediatric urinary tract infections caused by Escherichia coli and association with routine use of antibiotics in primary care: systematic review and meta-analysis. BMJ 352:i939. doi:10.1136/bmj.i939 26980184 PMC4793155

[21] Stewart CJ, Ajami NJ, O’Brien JL, Hutchinson DS, Smith DP, Wong MC, Ross MC, Lloyd RE, Doddapaneni H, Metcalf GA, Muzny D, Gibbs RA, Vatanen T, Huttenhower C, Xavier RJ, Rewers M, Hagopian W, Toppari J, Ziegler A-G, She J-X, Akolkar B, Lernmark A, Hyoty H, Vehik K, Krischer JP, Petrosino JF. 2018. Temporal development of the gut microbiome in early childhood from the TEDDY study. Nature 562:583–588. doi:10.1038/s41586-018-0617-x 30356187 PMC6415775

[22] Westerbeek EAM, van den Berg A, Lafeber HN, Knol J, Fetter WPF, van Elburg RM. 2006. The intestinal bacterial colonisation in preterm infants: a review of the literature. Clin Nutr 25:361–368. doi:10.1016/j.clnu.2006.03.002 16677741

[23] Thänert R, Sawhney SS, Schwartz DJ, Dantas G. 2022. The resistance within: antibiotic disruption of the gut microbiome and resistome dynamics in infancy. Cell Host Microbe 30:675–683. doi:10.1016/j.chom.2022.03.013 35550670 PMC9173668

[24] Cryan JF, O’Riordan KJ, Sandhu K, Peterson V, Dinan TG. 2020. The gut microbiome in neurological disorders. Lancet Neurol 19:179–194. doi:10.1016/S1474-4422(19)30356-4 31753762

[25] Nimer N, Choucair I, Wang Z, Nemet I, Li L, Gukasyan J, Weeks TL, Alkhouri N, Zein N, Tang WHW, Fischbach MA, Brown JM, Allayee H, Dasarathy S, Gogonea V, Hazen SL. 2021. Bile acids profile, histopathological indices and genetic variants for non-alcoholic fatty liver disease progression. Metabolism 116:154457. doi:10.1016/j.metabol.2020.154457 33275980 PMC7856026

[26] Lavelle A, Sokol H. 2020. Gut Microbiota-derived metabolites as key actors in inflammatory bowel disease. Nat Rev Gastroenterol Hepatol 17:223–237. doi:10.1038/s41575-019-0258-z 32076145

[27] Dominguez-Bello MG, Costello EK, Contreras M, Magris M, Hidalgo G, Fierer N, Knight R. 2010. Delivery mode shapes the acquisition and structure of the initial microbiota across multiple body habitats in newborns. Proc Natl Acad Sci U S A 107:11971–11975. doi:10.1073/pnas.1002601107 20566857 PMC2900693

[28] Gschwendtner S, Kang H, Thiering E, Kublik S, Fösel B, Schulz H, Krauss-Etschmann S, Heinrich J, Schöler A, Schloter M, Standl M. 2019. Early life determinants induce sustainable changes in the gut microbiome of six-year-old children. Sci Rep 9:12675. doi:10.1038/s41598-019-49160-7 31481742 PMC6722248

[29] Partridge SR, Kwong SM, Firth N, Jensen SO. 2018. Mobile genetic elements associated with antimicrobial resistance. Clin Microbiol Rev 31. doi:10.1128/CMR.00088-17 PMC6148190 30068738

[30] Juhas M. 2015. Horizontal gene transfer in human pathogens. Crit Rev Microbiol 41:101–108. doi:10.3109/1040841X.2013.804031 23862575

[31] Paramsothy S, Nielsen S, Kamm MA, Deshpande NP, Faith JJ, Clemente JC, Paramsothy R, Walsh AJ, van den Bogaerde J, Samuel D, Leong RWL, Connor S, Ng W, Lin E, Borody TJ, Wilkins MR, Colombel J-F, Mitchell HM, Kaakoush NO. 2019. Specific bacteria and metabolites associated with response to fecal microbiota transplantation in patients with ulcerative colitis. Gastroenterology 156:1440–1454. doi:10.1053/j.gastro.2018.12.001 30529583

[32] Mahmoodi F, Rezatofighi SE, Akhoond MR. 2020. Antimicrobial resistance and metallo-beta-lactamase producing among commensal Escherichia coli isolates from healthy children of Khuzestan and Fars provinces; Iran. BMC Microbiol 20:366. doi:10.1186/s12866-020-02051-8 33256594 PMC7708168

[33] Li X, Stokholm J, Brejnrod A, Vestergaard GA, Russel J, Trivedi U, Thorsen J, Gupta S, Hjelmsø MH, Shah SA, Rasmussen MA, Bisgaard H, Sørensen SJ. 2021. The infant gut resistome associates with E. coli, environmental exposures, gut microbiome maturity, and asthma-associated bacterial composition. Cell Host & Microbe 29:975–987. doi:10.1016/j.chom.2021.03.017 33887206

[34] Olm MR, Dahan D, Carter MM, Merrill BD, Yu FB, Jain S, Meng X, Tripathi S, Wastyk H, Neff N, Holmes S, Sonnenburg ED, Jha AR, Sonnenburg JL. 2022. Robust variation in infant gut microbiome assembly across a spectrum of lifestyles. Science 376:1220–1223. doi:10.1126/science.abj2972 35679413 PMC9894631

[35] Weersma RK, Zhernakova A, Fu J. 2020. Interaction between drugs and the gut Microbiome. Gut 69:1510–1519. doi:10.1136/gutjnl-2019-320204 32409589 PMC7398478

[36] Bonder MJ, Kurilshikov A, Tigchelaar EF, Mujagic Z, Imhann F, Vila AV, Deelen P, Vatanen T, Schirmer M, Smeekens SP, et al.. 2016. The effect of host genetics on the gut microbiome. Nat Genet 48:1407–1412. doi:10.1038/ng.3663 27694959

[37] Liu J, Taft DH, Maldonado-Gomez MX, Johnson D, Treiber ML, Lemay DG, DePeters EJ, Mills DA. 2019. The fecal resistome of dairy cattle is associated with diet during nursing. Nat Commun 10:4406. doi:10.1038/s41467-019-12111-x 31562300 PMC6765000

[38] Stearns JC, Zulyniak MA, de Souza RJ, Campbell NC, Fontes M, Shaikh M, Sears MR, Becker AB, Mandhane PJ, Subbarao P, Turvey SE, Gupta M, Beyene J, Surette MG, Anand SS, NutriGen Alliance. 2017. Ethnic and diet-related differences in the healthy infant microbiome. Genome Med 9:32. doi:10.1186/s13073-017-0421-5 28356137 PMC5372248

[39] Bokulich NA, Chung J, Battaglia T, Henderson N, Jay M, Li H, D Lieber A, Wu F, Perez-Perez GI, Chen Y, Schweizer W, Zheng X, Contreras M, Dominguez-Bello MG, Blaser MJ. 2016. Antibiotics, birth mode, and diet shape microbiome maturation during early life. Sci Transl Med 8:343ra82. doi:10.1126/scitranslmed.aad7121 PMC5308924 27306664

[40] Nolivos S, Cayron J, Dedieu A, Page A, Delolme F, Lesterlin C. 2019. Role of AcrAB-TolC multidrug efflux pump in drug-resistance acquisition by plasmid transfer. Science 364:778–782. doi:10.1126/science.aav6390 31123134

[41] Tortoli E. 2014. Microbiological features and clinical relevance of new species of the genus Mycobacterium. Clin Microbiol Rev 27:727–752. doi:10.1128/CMR.00035-14 25278573 PMC4187642

[42] Nessar R, Cambau E, Reyrat JM, Murray A, Gicquel B. 2012. Mycobacterium abscessus: a new antibiotic nightmare. J Antimicrob Chemother 67:810–818. doi:10.1093/jac/dkr578 22290346

[43] Liu Z, Klümper U, Liu Y, Yang Y, Wei Q, Lin J-G, Gu J-D, Li M. 2019. Metagenomic and metatranscriptomic analyses reveal activity and hosts of antibiotic resistance genes in activated sludge. Environ Int 129:208–220. doi:10.1016/j.envint.2019.05.036 31129497

[44] Sorek R, Cossart P. 2010. Prokaryotic transcriptomics: a new view on regulation, physiology and pathogenicity. Nat Rev Genet 11:9–16. doi:10.1038/nrg2695 19935729

[45] Ewels P, Magnusson M, Lundin S, Käller M. 2016. Multiqc: summarize analysis results for multiple tools and samples in a single report. Bioinformatics 32:3047–3048. doi:10.1093/bioinformatics/btw354 27312411 PMC5039924

[46] Segata N, Waldron L, Ballarini A, Narasimhan V, Jousson O, Huttenhower C. 2012. Metagenomic microbial community profiling using unique clade-specific marker genes. Nat Methods 9:811–814. doi:10.1038/nmeth.2066 22688413 PMC3443552

[47] Franzosa EA, McIver LJ, Rahnavard G, Thompson LR, Schirmer M, Weingart G, Lipson KS, Knight R, Caporaso JG, Segata N, Huttenhower C. 2018. Species-level functional profiling of metagenomes and metatranscriptomes. Nat Methods 15:962–968. doi:10.1038/s41592-018-0176-y 30377376 PMC6235447

[48] Li D, Liu C-M, Luo R, Sadakane K, Lam T-W. 2015. MEGAHIT: an ultra-fast single-node solution for large and complex metagenomics assembly via succinct de Bruijn graph. Bioinformatics 31:1674–1676. doi:10.1093/bioinformatics/btv033 25609793

[49] Hyatt D, Chen G-L, Locascio PF, Land ML, Larimer FW, Hauser LJ. 2010. Prodigal: prokaryotic gene recognition and translation initiation site identification. BMC Bioinformatics 11:119. doi:10.1186/1471-2105-11-119 20211023 PMC2848648

[50] Huang Y, Niu B, Gao Y, Fu L, Li W. 2010. CD-HIT suite: a web server for clustering and comparing biological sequences. Bioinformatics 26:680–682. doi:10.1093/bioinformatics/btq003 20053844 PMC2828112

[51] Wood DE, Salzberg SL. 2014. Kraken: ultrafast metagenomic sequence classification using exact alignments. Genome Biol 15:R46. doi:10.1186/gb-2014-15-3-r46 24580807 PMC4053813

[52] Alcock BP, Raphenya AR, Lau TTY, Tsang KK, Bouchard M, Edalatmand A, Huynh W, Nguyen A-L, Cheng AA, Liu S, et al.. 2020. CARD 2020: antibiotic resistome surveillance with the comprehensive antibiotic resistance database. Nucleic Acids Res 48:D517–D525. doi:10.1093/nar/gkz935 31665441 PMC7145624

[53] Buchfink B, Xie C, Huson DH. 2015. Fast and sensitive protein alignment using DIAMOND. Nat Methods 12:59–60. doi:10.1038/nmeth.3176 25402007

[54] Li Z, Shi L, Wang B, Wei X, Zhang J, Guo T, Kong J, Wang M, Xu H. 2021. In vitro assessment of antimicrobial resistance dissemination dynamics during multidrug-resistant-bacterium invasion events by using a continuous-culture device. Appl Environ Microbiol 87:e02659-20. doi:10.1128/AEM.02659-20 33361364 PMC8104991

