# Supplementary figures and images for: Temporal development and potential interactions between the gut microbiome and resistome in early childhood
Source: Microbiol Spectr. 2024 Jan 9;12(2):e03177-23. doi: 10.1128/spectrum.03177-23 (PMC10846076; doi:10.1128/spectrum.03177-23)

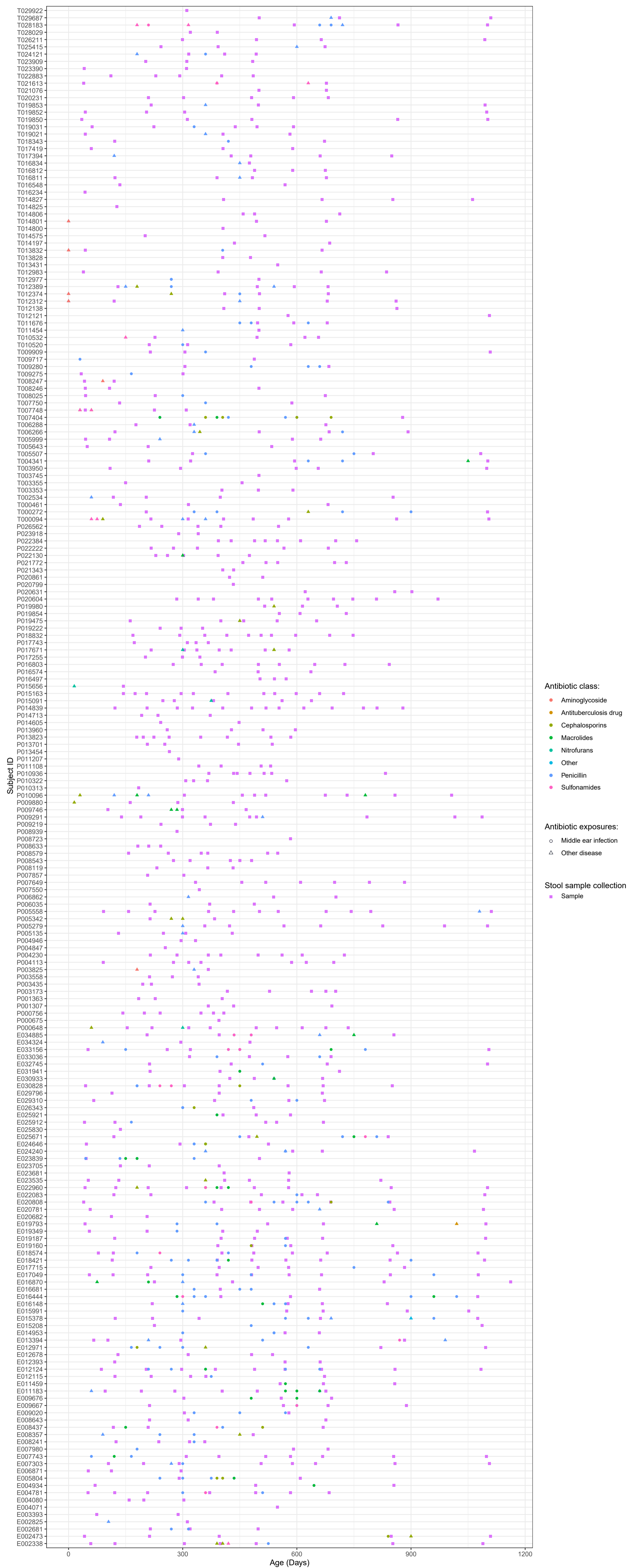

Figure S8 The information of samples and antibiotic exposure in the cohort

Supplement: Figure S8 — The information of samples and antibiotic exposure in the cohort. [file spectrum.03177-23-s0001.pdf]
