# Supplementary material for: Temporal development and potential interactions between the gut microbiome and resistome in early childhood
Source: Microbiol Spectr. 2024 Jan 9;12(2):e03177-23. doi: 10.1128/spectrum.03177-23 (PMC10846076; doi:10.1128/spectrum.03177-23)
Supplement: Supplemental material — Figures S1 to S23; Table S4. [file spectrum.03177-23-s0002.pdf]

SUPPLEMENTAL MATERIAL

Contents

Figure S1 3

Figure S2 3

Figure S3 4

Figure S4 5

Figure S5 6

Figure S6 7

Figure S7 8

Figure S9 9

Figure S10 10

Figure S11 11

Figure S12 12

Figure S13 13

Figure S14 14

Figure S16 15

Figure S17 16

Figure S18 17

Figure S19 18

Figure S20 19

Figure S21 20

Figure S22 20

Figure S23 21

Table S4 22

Figures

Figure S1 Effects of country on gut microbiome and resistome in children. β-diversity analysis and PERMANOVA of (a) gut resisitome and (b) gut microbiome.

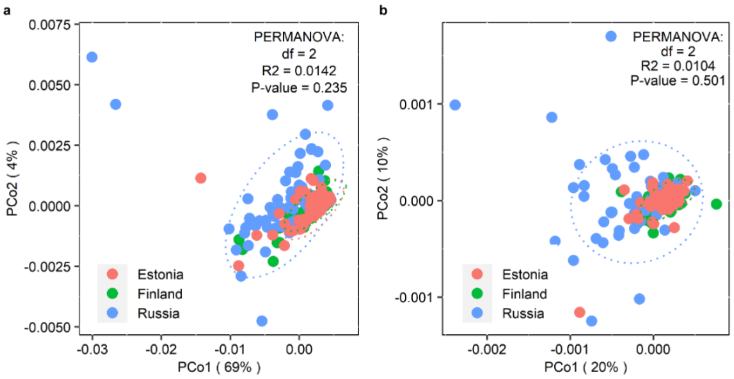

Figure S2 *In vitro* experiments to investigate the proportion of antibiotic-resistant bacteria in *E. coli* flora under ampicillin pressure.

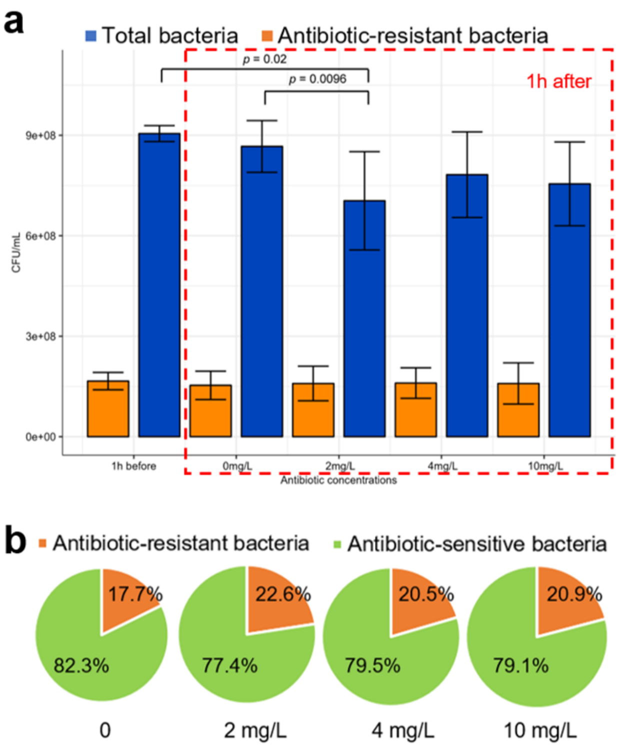

Figure S3 The estimated variance in stability index per child in gut microbiome and resistome. The estimated variance of (a) gut microbiota and (d) gut resistome for children of “With Antibiotics” and “Without Antibiotics” (mean ± SE) with at least two samples per child. The estimated variance of (b) gut microbiota and (e) gut resistome for children of “With Antibiotics” and “Without Antibiotics” (mean ± SE) with at least three samples in different age groups per child. The estimated variance of (c) gut microbiota and (f) gut resistome for children of “With Antibiotics” and “Without Antibiotics” (mean ± SE) with at least three samples in different age groups per child and number of antibiotic treatments no less than six in “With Antibiotics” children.

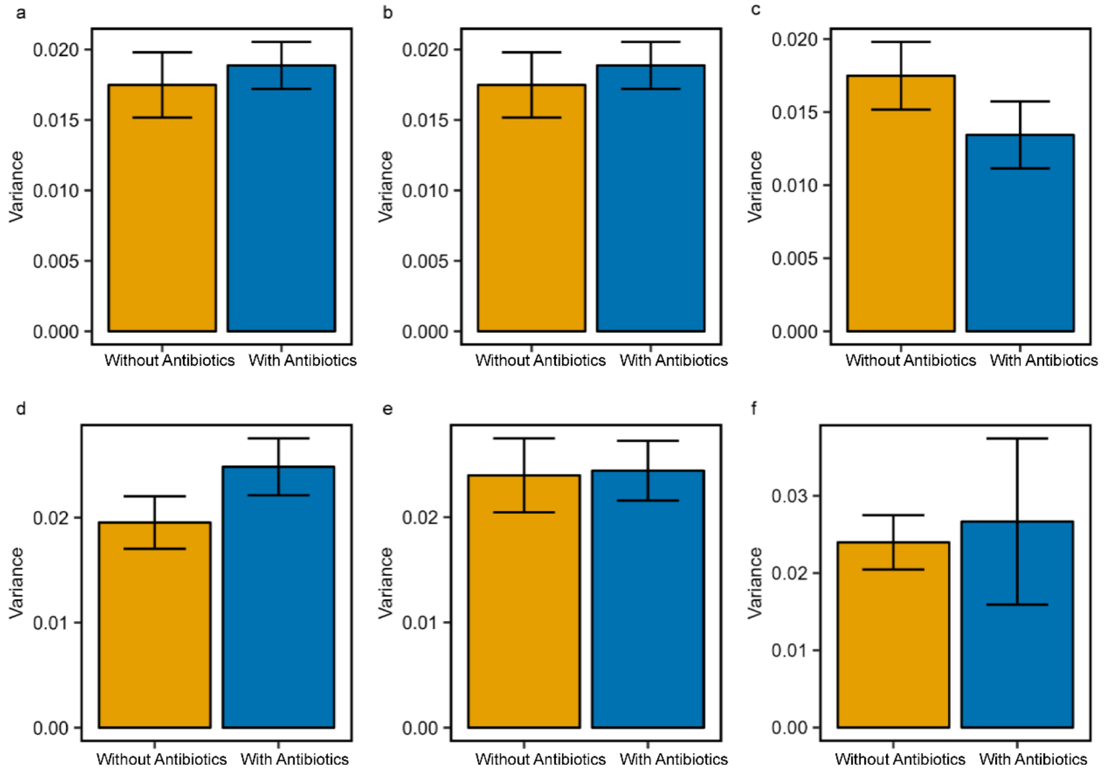

Figure S4 Effects of antibiotic use on stability of microbipme and antibiotic resistome. Comparison of (a) gut resistome and gut microbiota (b) stability in children of "With Antibiotics" and "Without Antibiotics" with samples at different age groups (0-6, 6-12, and 18-36 month) per child. *:*P* <=0.05, NA: *P* >0.05.

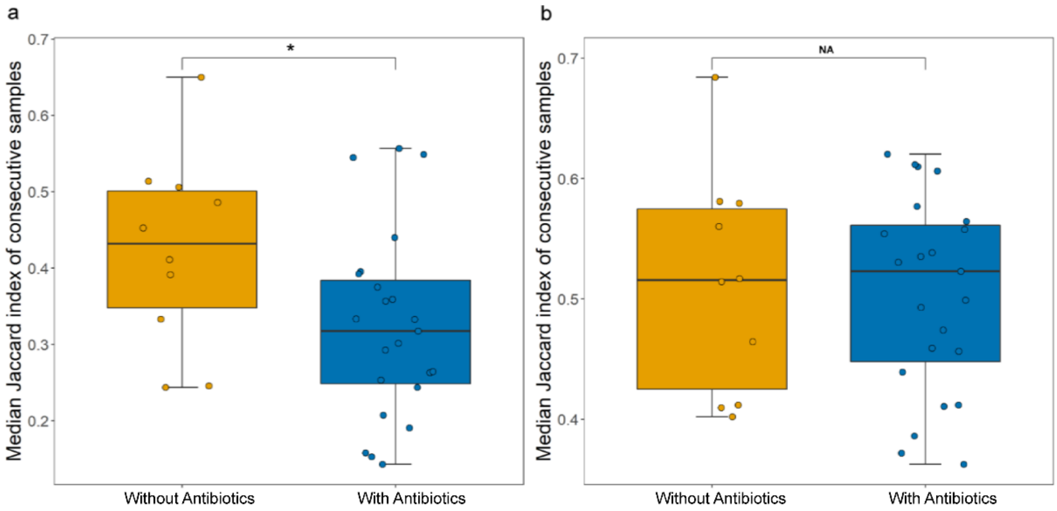

Figure S5 Development of ARGs belonging to antibiotic inactivation of children in the "With Antibiotics" group. Longitudinal changes (a) and proportion at age three (b) of ARGs belonging to antibiotic inactivation in the "With Antibiotics" children.

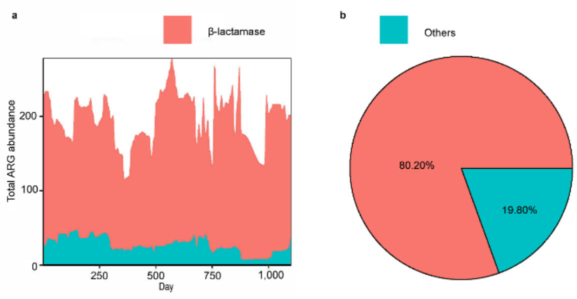

Figure S6 Average composition of gut microbiome of "With Antibiotics"(a) and "Without Antibiotics" (b) children during the first three years of age.

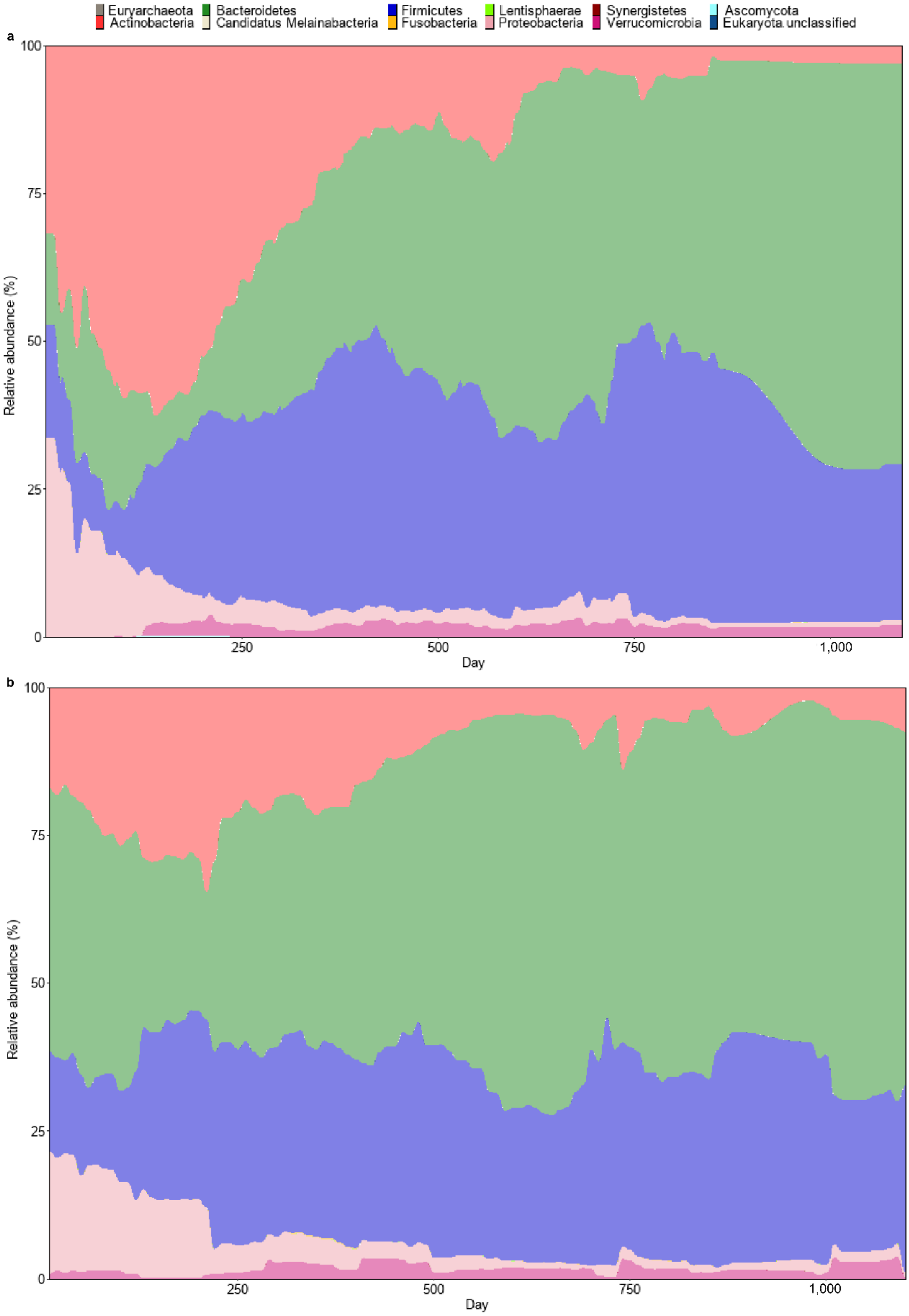

Figure S7 The realstionship of ARGs mechanism and species. (a) The mechanism of ARGs and the corresponding species were displayed; (b) A map showing antibiotic efflux pump genes from E. coli and other species;(c) The proportion of E. coli with efflux genes in different age groups.

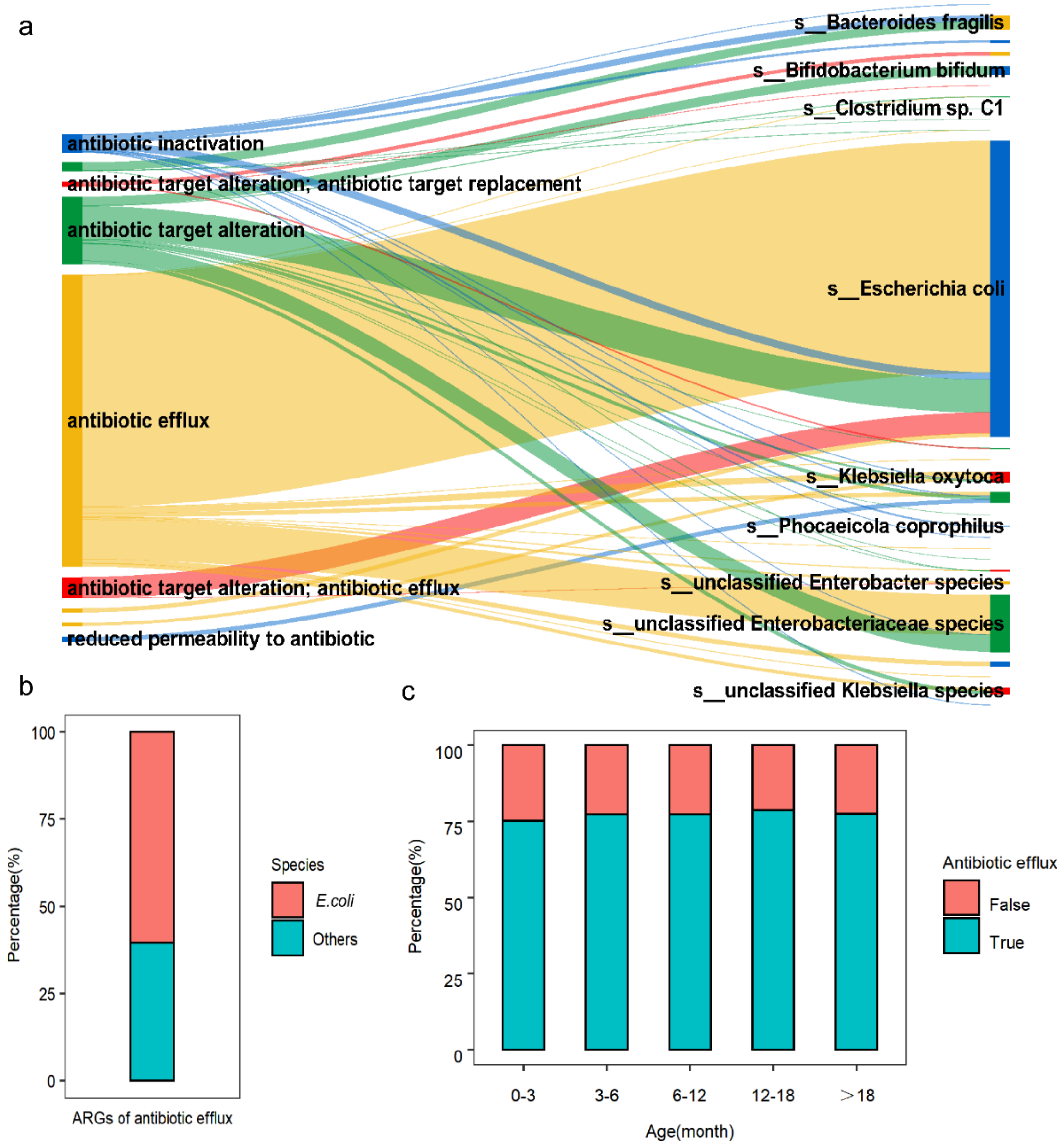

Figure S9 ARG, species, and average phylum-level composition profiles of individual (E022960). (a) Abundance of ARG (TPM: Transcripts Per Kilobase Million) over time, together with the timing of individual antibiotic courses (colored dots) and diseases (colored triangles). (b) Relative abundance of species that most correlated with the ARG profiles. (c) Average phylum-level composition.

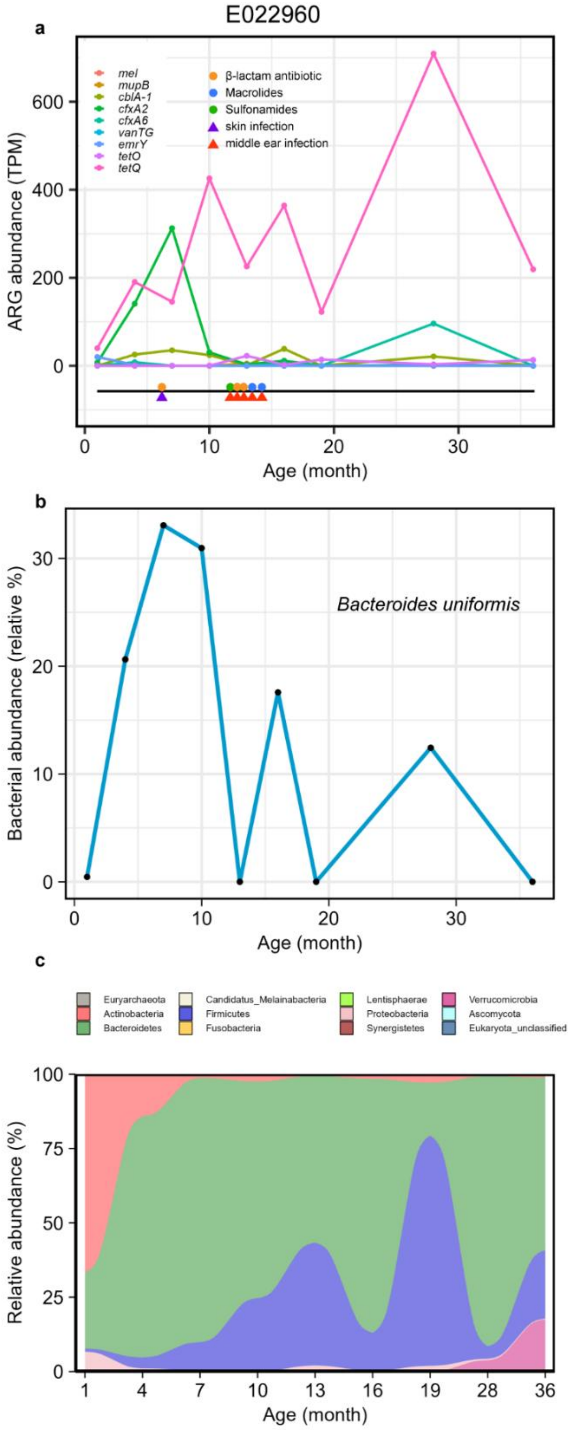

Figure S10 ARG richness among bacterial species and phyla in 0-3 months.

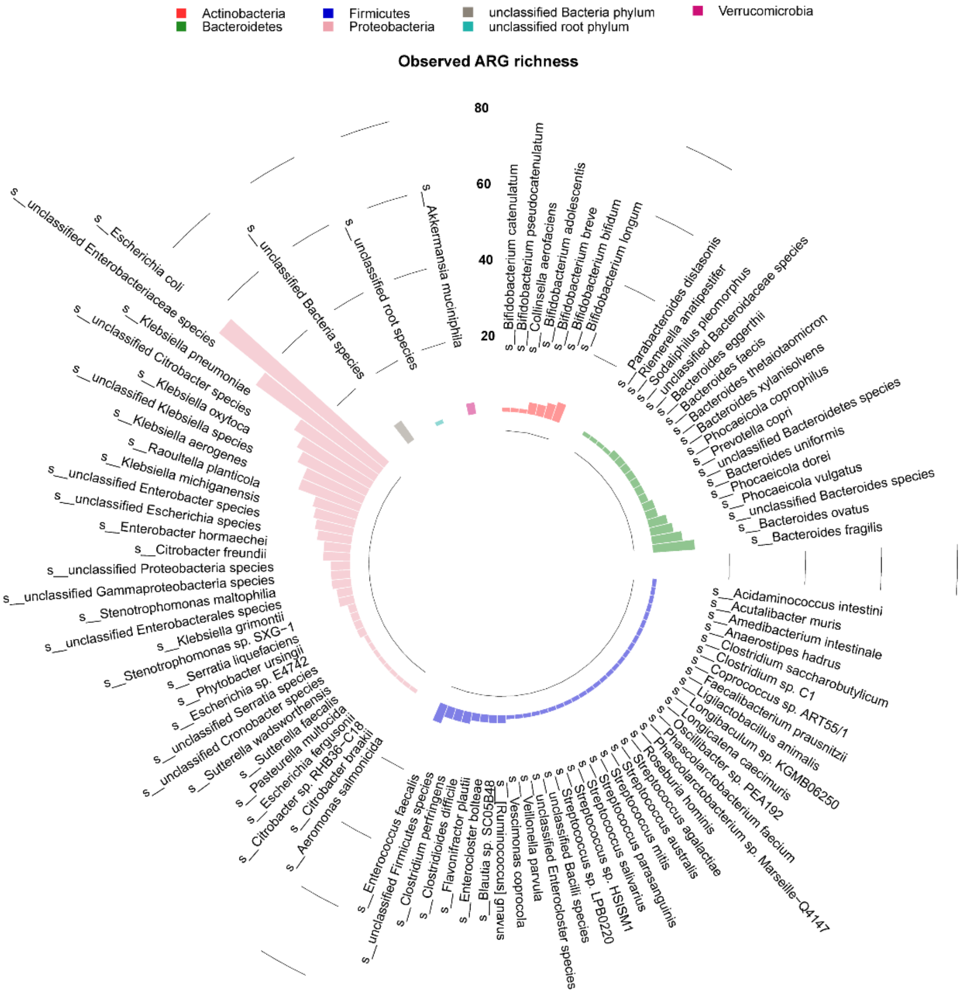

Figure S11 ARG richness among bacterial species and phyla in 3-6 months.

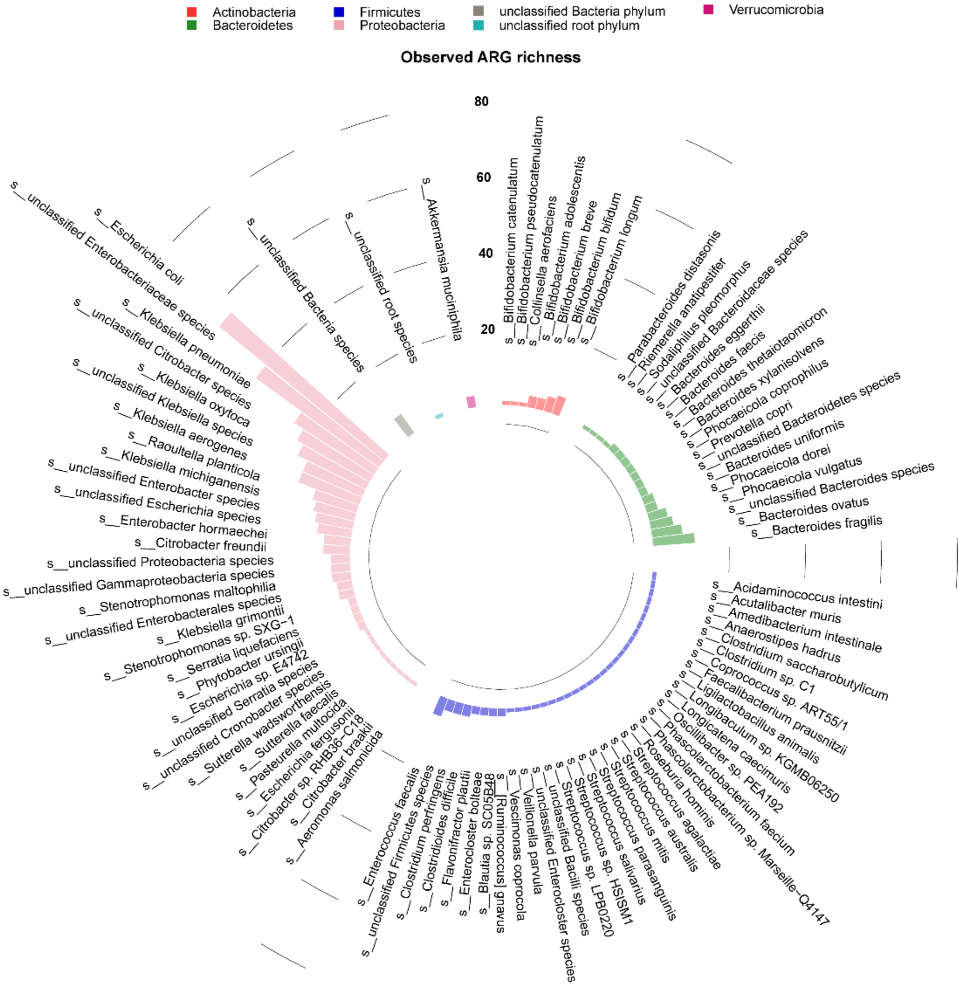

Figure S12 ARG richness among bacterial species and phyla in 6-12 months.

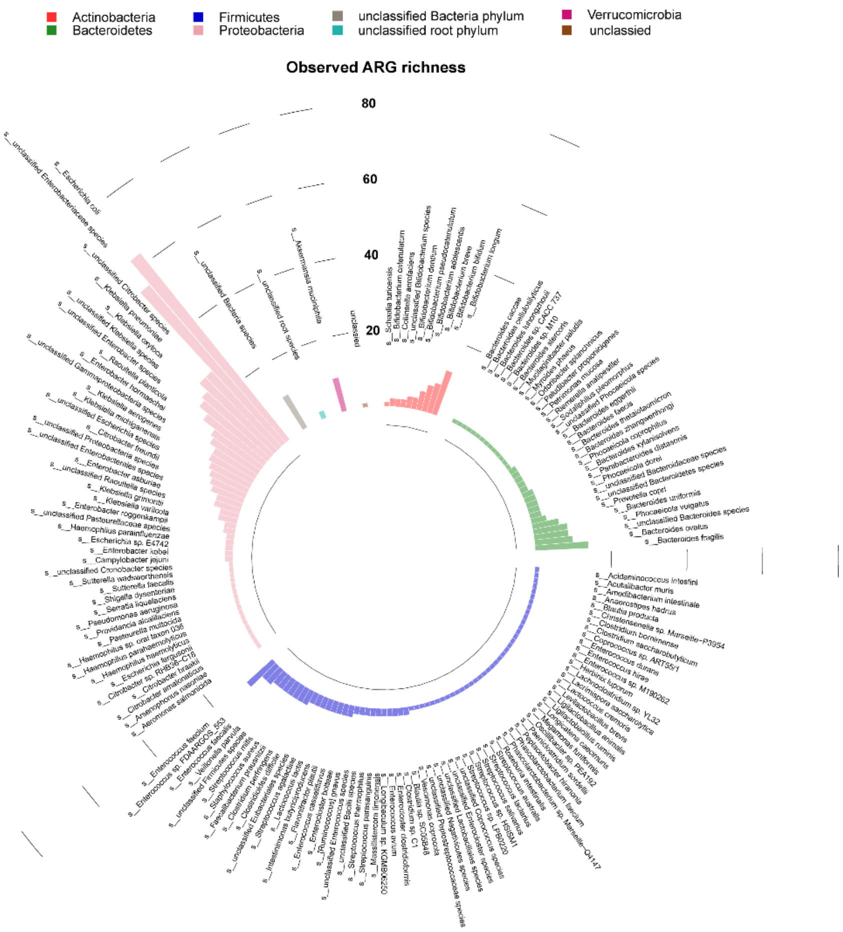

Figure S13 ARG richness among bacterial species and phyla in 12-18 months.

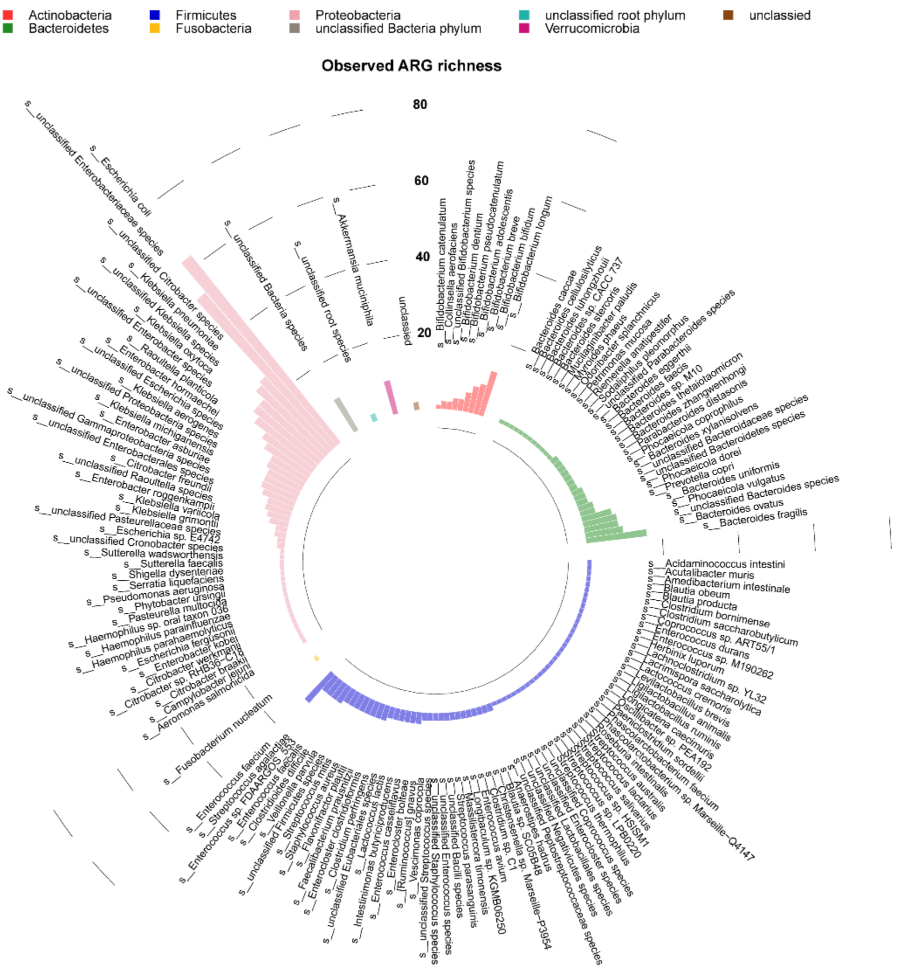

Figure S14 ARG richness among bacterial species and phyla in 18-36 months.

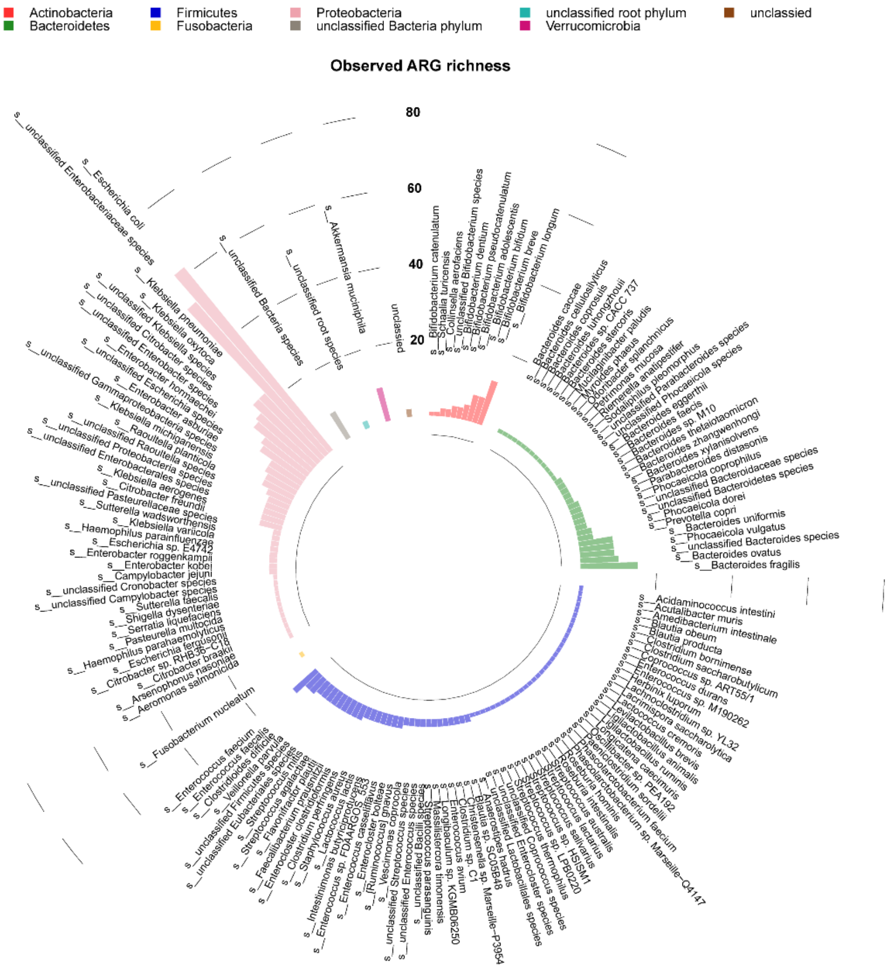

Figure S15 Average phylum-level ARGs richness during five age groups.

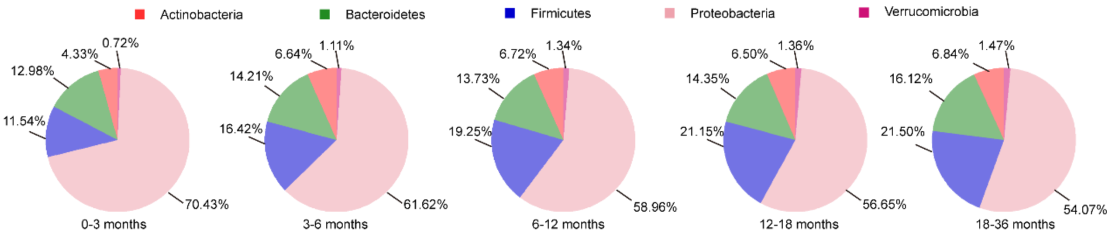

Figure S16 Log-transformed total ARG abundance among bacterial species and phyla in 0-3 months.

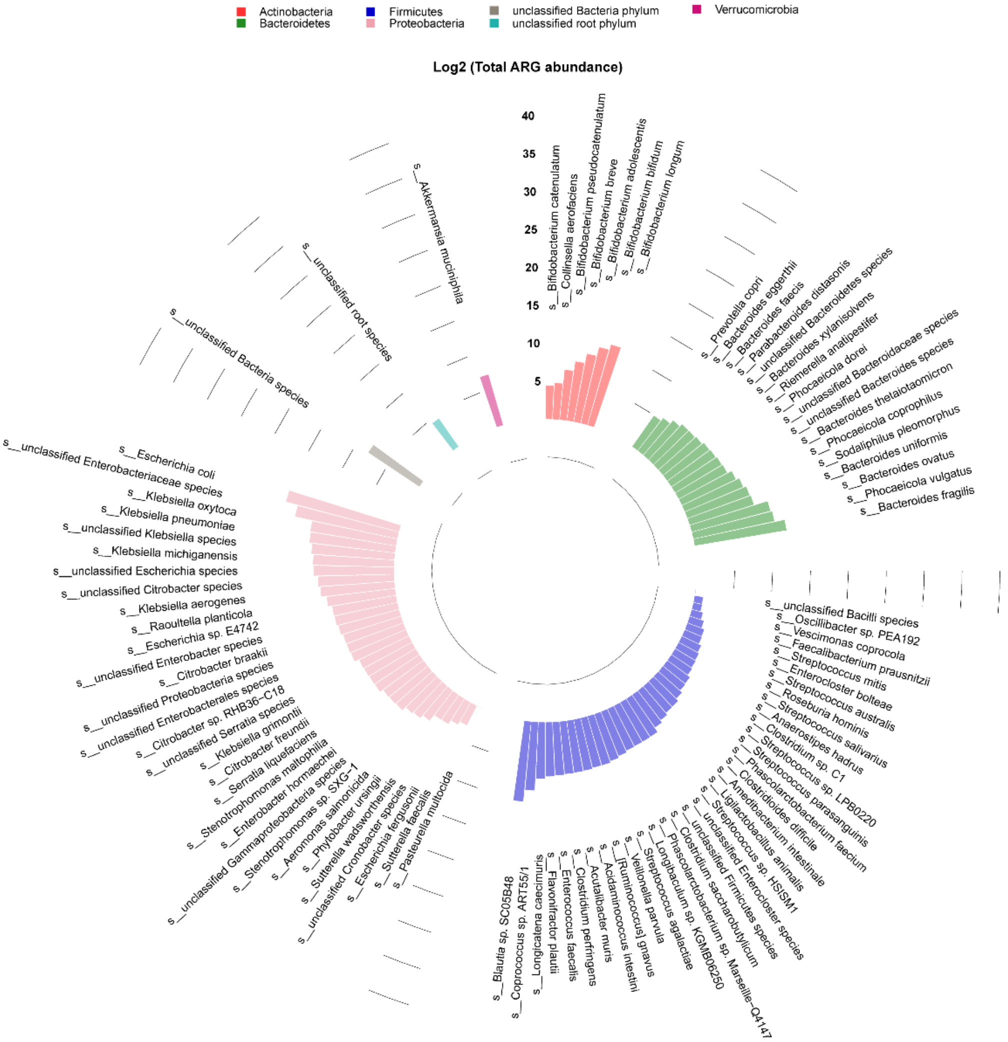

Figure S17 Log-transformed total ARG abundance among bacterial species and phyla in 3-6 months.

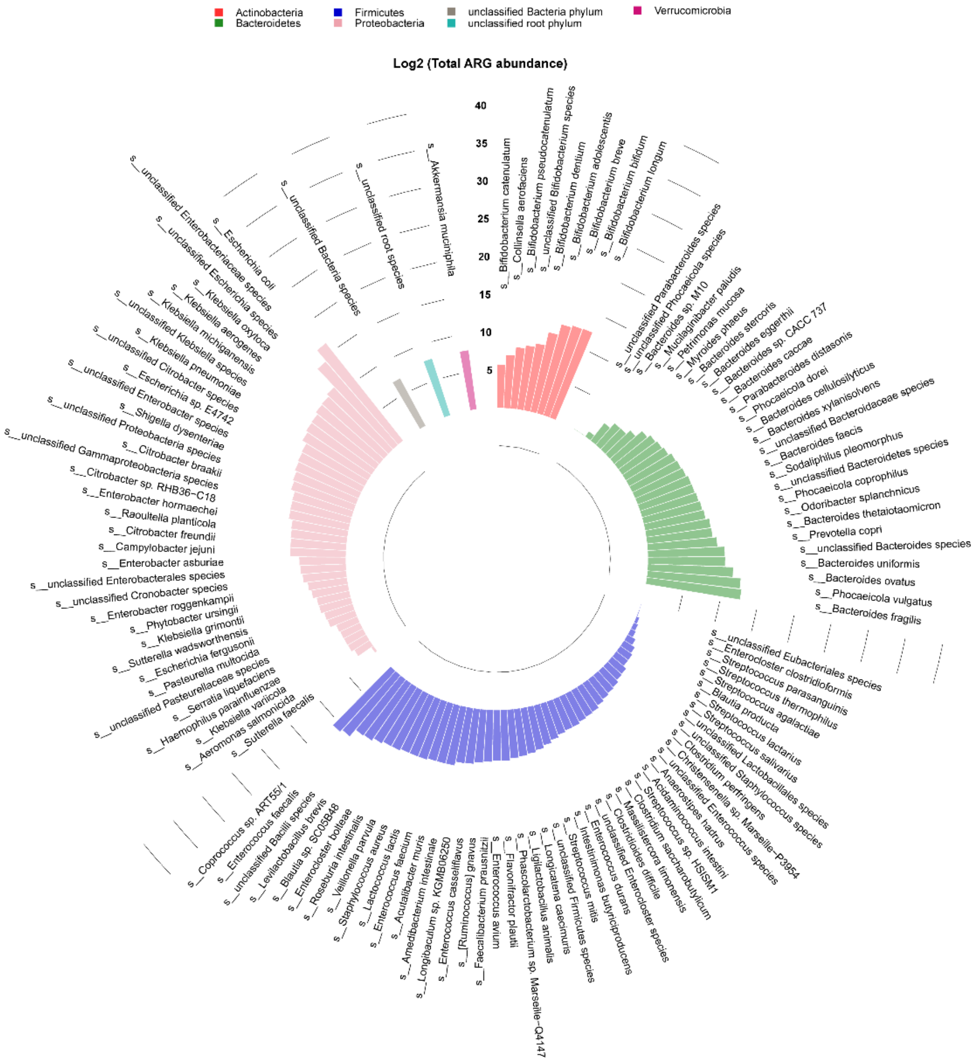

Figure S18 Log-transformed total ARG abundance among bacterial species and phyla in 6-12 months.

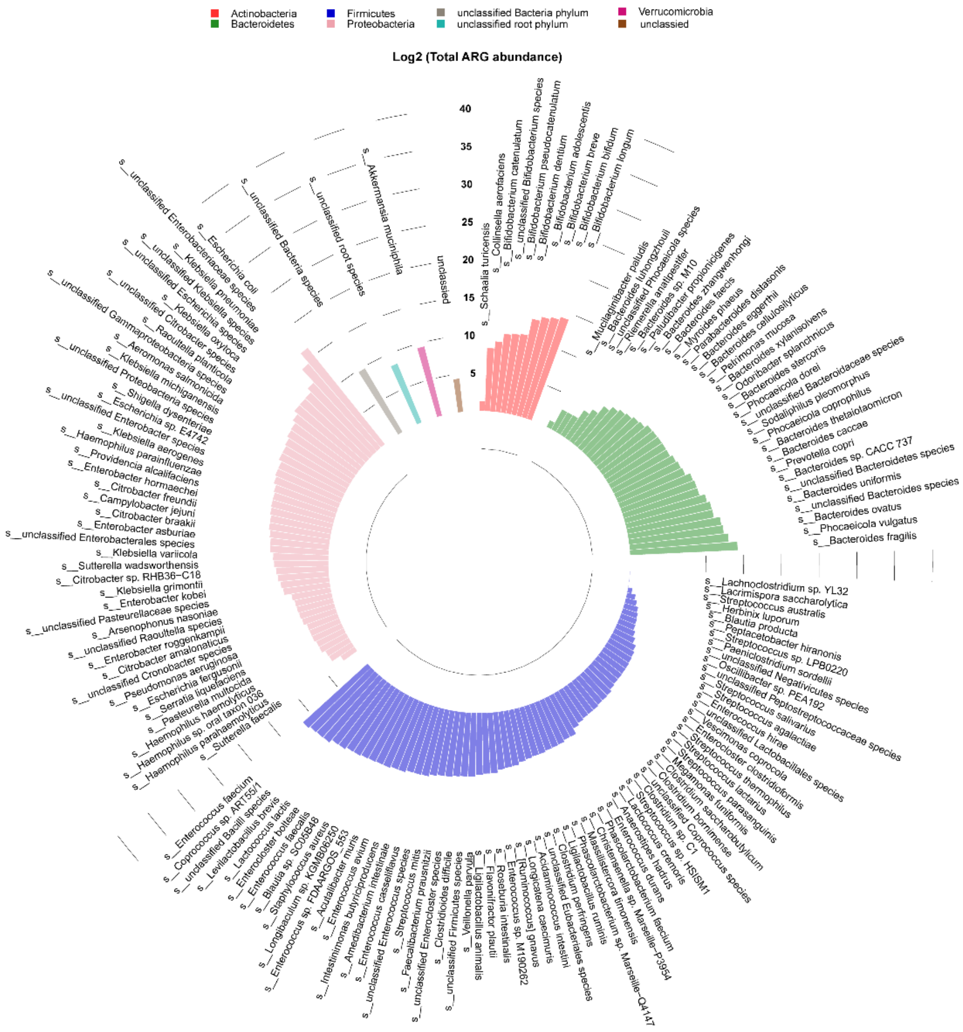

Figure S19 Log-transformed total ARG abundance among bacterial species and phyla in 12-18 months.

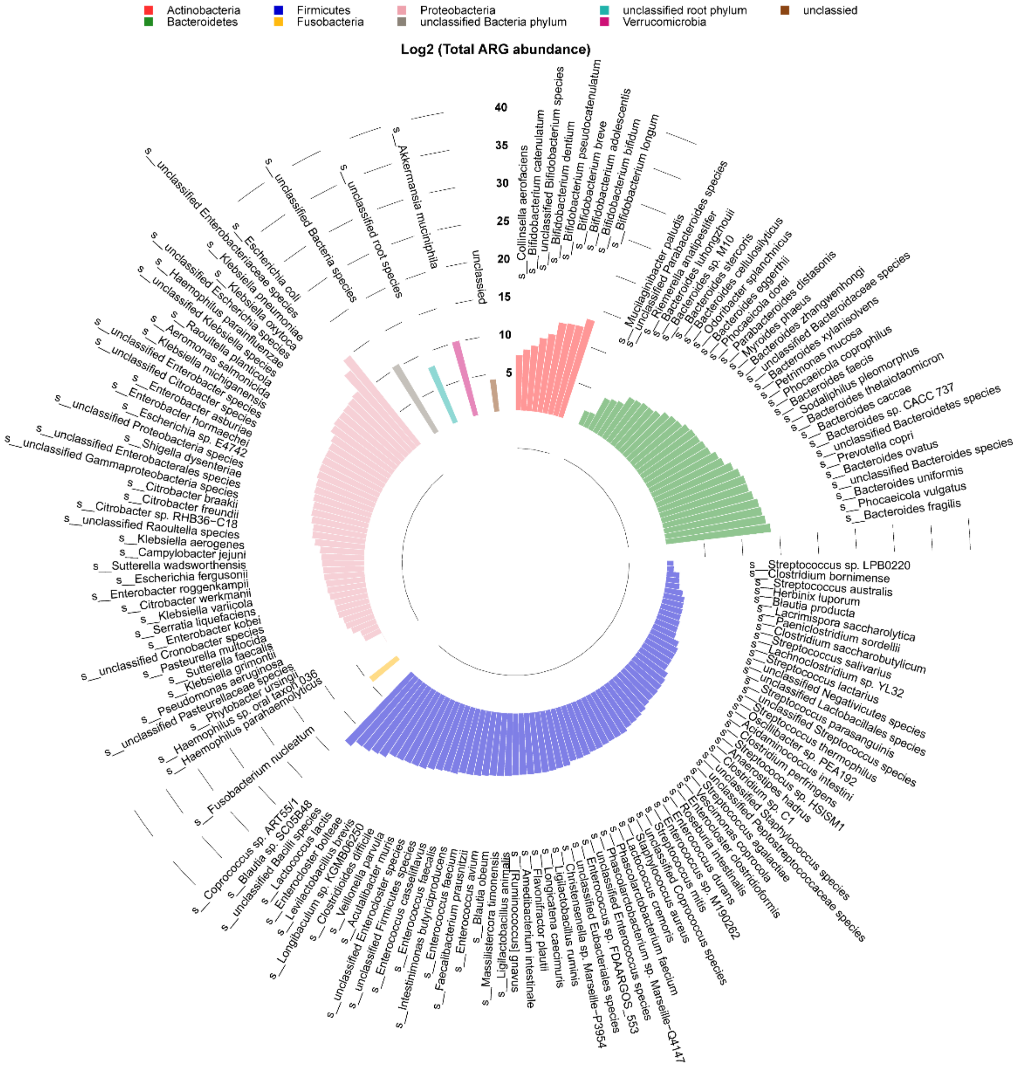

Figure S20 Log-transformed total ARG abundance among bacterial species and phyla in 18-36 months.

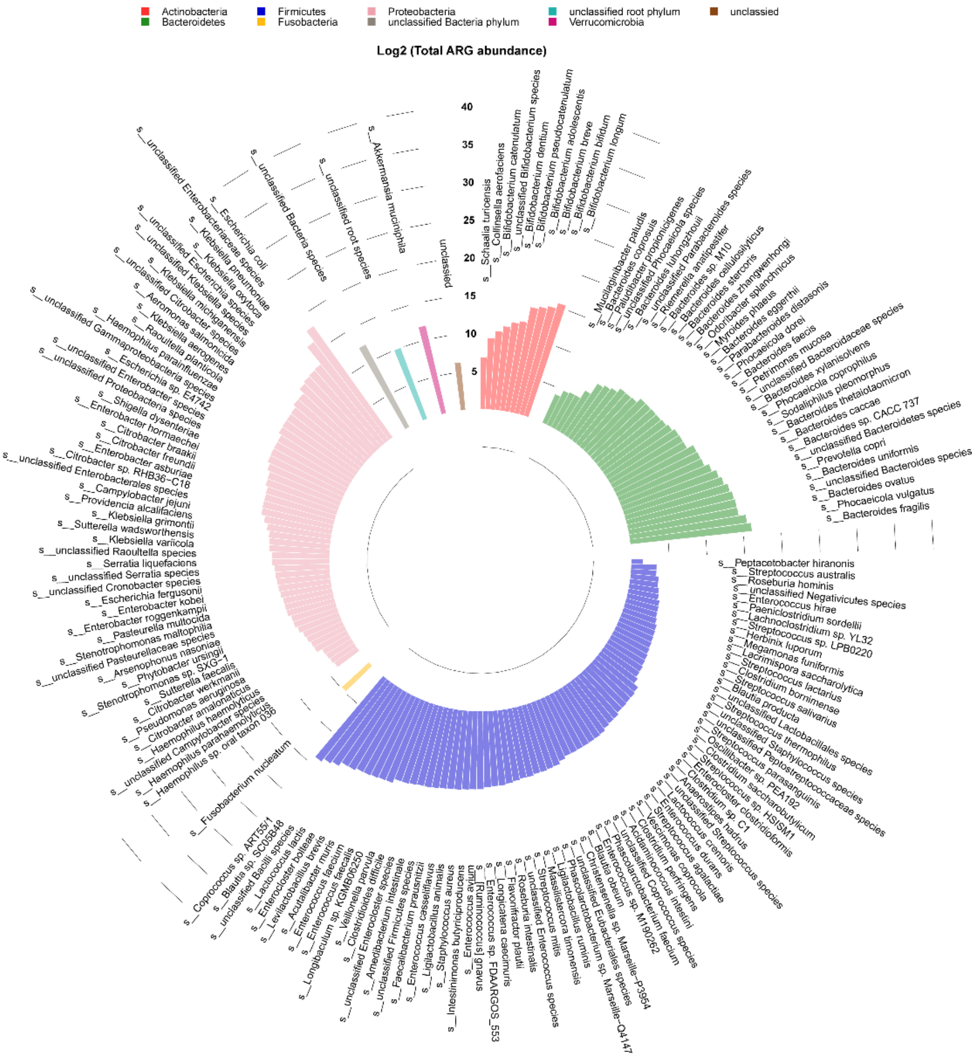

Figure S21 Log-transformed total ARG abundance among bacterial species and phyla.

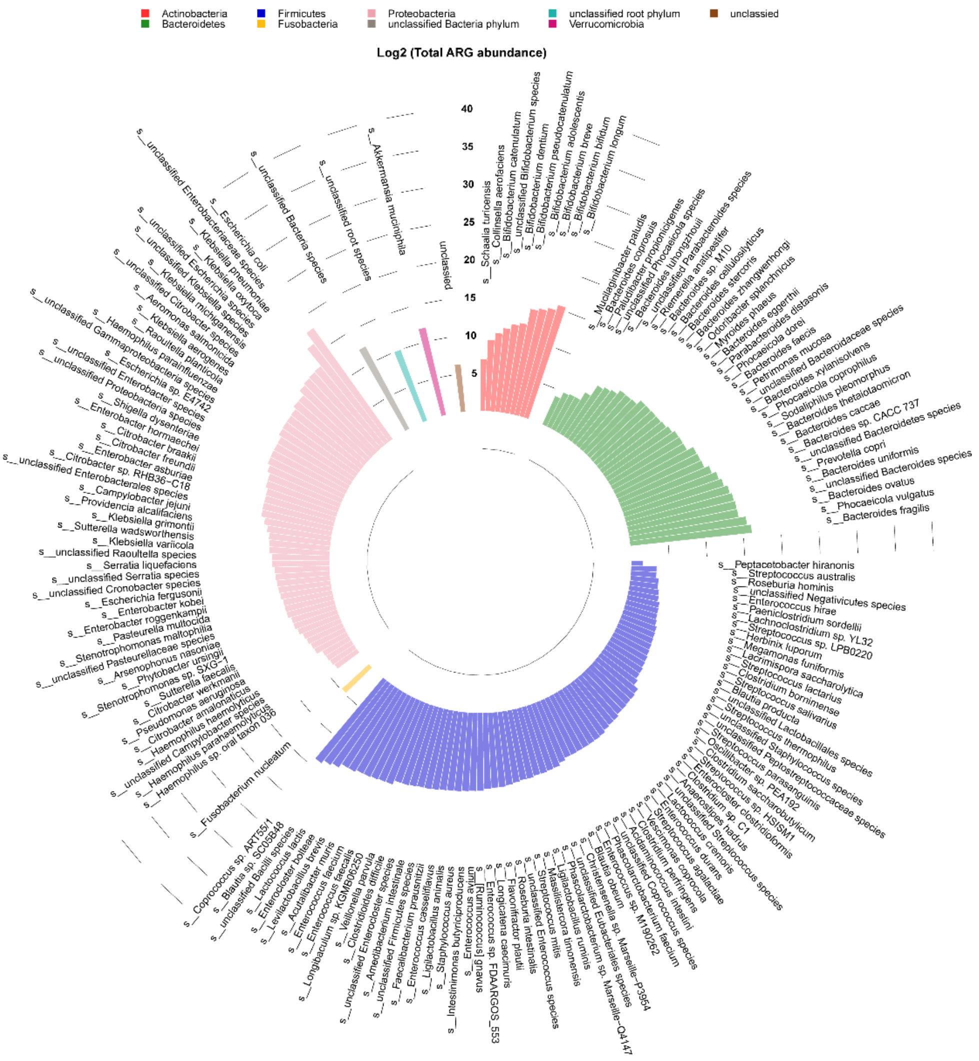

Figure S22 Average phylum-level ARGs abundance during five age groups.

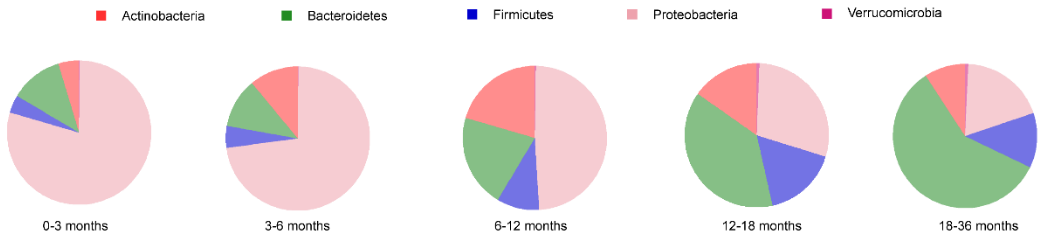

Figure S23 Ecological network of bacteria and ARGs. Correlations between ARGs and (a) *E. coli* or (b) *Klebsiella* were calculated through Spearman's rank correlation analysis. Only statistically significant correlations ($P < 0.05$) with $r > 0.5$ were plotted. The color of the node represents the ARGs of different antibiotic resistance mechanisms or *species*.

a

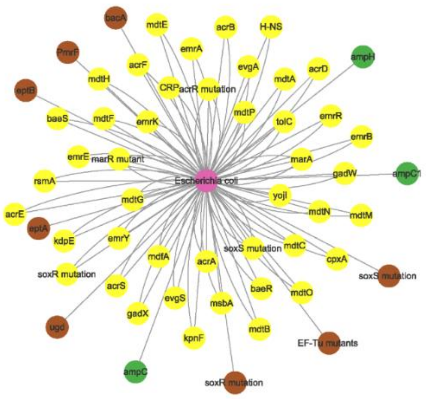

b

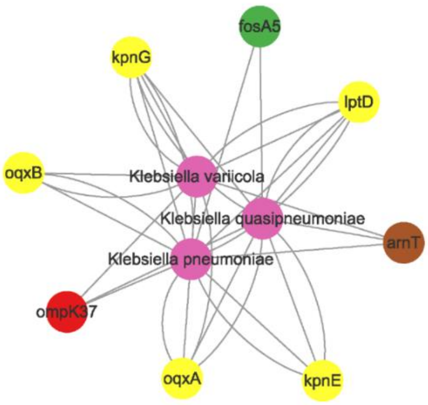

Tables

Table S1 Types of antibiotics used, duration, and the specific clinical disease for antibiotics use of 118 'With Antibiotics' children.

Table S2 Maaslin result of total samples.

Table S3 Mediation result with acem value greater than zero and p value less than 0.05.

Table S4 The Procrustes analysis result of correlation between the gut microbiome and resistome for each age group.

Age group	correlation coefficient r	*P* value
0-3 months	0.981	0.759
**3-6 months**	**0.91**	**0.014**
6-12 months	0.999	0.948
12-18 months	0.987	0.124
18-36 months	0.999	0.882
